# Online neurostimulation of Broca’s area does not interfere with syntactic predictions: A combined TMS-EEG approach to basic linguistic combination

**DOI:** 10.3389/fpsyg.2022.968836

**Published:** 2022-10-28

**Authors:** Matteo Maran, Ole Numssen, Gesa Hartwigsen, Emiliano Zaccarella

**Affiliations:** ^1^Department of Neuropsychology, Max Planck Institute for Human Cognitive and Brain Sciences, Leipzig, Germany; ^2^International Max Planck Research School on Neuroscience of Communication: Function, Structure, and Plasticity, Leipzig, Germany; ^3^Lise Meitner Research Group Cognition and Plasticity, Max Planck Institute for Human Cognitive and Brain Sciences, Leipzig, Germany

**Keywords:** Broca’s area, syntax, prediction, two-word, TMS-EEG, EEG, TMS, phrase

## Abstract

Categorical predictions have been proposed as the key mechanism supporting the fast pace of syntactic composition in language. Accordingly, grammar-based expectations are formed—e.g., the determiner “a” triggers the prediction for a noun—and facilitate the analysis of incoming syntactic information, which is then checked against a single or few other word categories. Previous functional neuroimaging studies point towards Broca’s area in the left inferior frontal gyrus (IFG) as one fundamental cortical region involved in categorical prediction during incremental language processing. Causal evidence for this hypothesis is however still missing. In this study, we combined Electroencephalography (EEG) and Transcranial Magnetic Stimulation (TMS) to test whether Broca’s area is functionally relevant in predictive mechanisms for language. We transiently perturbed Broca’s area during the first word in a two-word construction, while simultaneously measuring the Event-Related Potential (ERP) correlates of syntactic composition. We reasoned that if Broca’s area is involved in predictive mechanisms for syntax, disruptive TMS during the first word would mitigate the difference in the ERP responses for predicted and unpredicted categories in basic two-word constructions. Contrary to this hypothesis, perturbation of Broca’s area at the predictive stage did not affect the ERP correlates of basic composition. The correlation strength between the electrical field induced by TMS and the ERP responses further confirmed this pattern. We discuss the present results considering an alternative account of the role of Broca’s area in syntactic composition, namely the bottom-up integration of words into constituents, and of compensatory mechanisms within the language predictive network.

## Introduction

The combination of words into larger units is a hallmark of the human language faculty. A compositional engine overcomes the size of the lexicon, making it possible to convey an infinite number of meanings from a limited set of words. Syntactic rules are at the basis of this process, binding words into hierarchically structured phrases and sentences according to grammatical categorical information (Chomsky, 1995; Berwick et al., 2013; Everaert et al., 2015; Friederici et al., 2017).

At the neural level, the analysis of grammatical category is prioritized over other linguistic information (Friederici, 2011), mirroring the central role of syntactic composition in language. This is reflected in the earliness of the Event-Related Potential (ERP) components elicited by syntactic categorical violations (e.g., *the forget^1^), such as the Early Left Anterior Negativity (ELAN, Neville et al., 1991; Friederici et al., 1993), the Early Syntactic Negativity (ESN, Hasting and Kotz, 2008) and the Syntactic Mismatch Negativity (sMMN, Hasting et al., 2007; Herrmann et al., 2009). The latencies of these components show that categorical analysis occurs in approximately 200–250 milliseconds (ms), preceding thematic and semantic relations (Friederici, 2011). This first step of syntactic analysis occurs in a highly automatic fashion, as the sMMN and ESN effects are also elicited in the presence of distracting conditions (Hasting et al., 2007; Hasting and Kotz, 2008; Herrmann et al., 2009; Batterink and Neville, 2013). Moreover, the ELAN is not influenced by task-specific strategies or the probability of violation occurrence (Hahne and Friederici, 1999, 2002).

The earliness of categorical analysis has been proposed to rely on syntactic predictive mechanisms (Lau et al., 2006; Dikker et al., 2009, 2010; Jakuszeit et al., 2013). According to this hypothesis, syntactic predictions restrict the grammatical information to be checked against a target category (e.g., *The* → prediction for a noun) and allow fast analysis of incoming words. Computationally, this idea is reminiscent of left-corner parsing models (Abney and Johnson, 1991; Resnik, 1992; Hale, 2014) which perform incremental syntactic analysis by opening a phrase as soon as its leftmost element is encountered (e.g., *The* → opening of a determiner phrase and prediction for a noun). Once a constituent (e.g., a determiner phrase) is opened based on its left-most element (e.g., determiner), the parser incrementally predicts the category (e.g., a noun) of the upcoming word before it is actually encountered. Crucially, this prediction is based on syntax, as the expected grammatical category allows to close the open constituent (Ferreira and Qiu, 2021). At the neural level, this hypothesis is grounded on the assumption that the brain minimizes the processing load of incoming input by top-down predictions, which are passed from higher to lower levels of the functional architecture (Rao and Ballard, 1999; Friston, 2003; Friston and Kiebel, 2009). When the current input does not correspond to the expected one, a mismatch signal (i.e., the prediction error) is generated and the internal model is updated (Friston, 2005; Garrido et al., 2007; Den Ouden H. E. M. et al., 2012). Accordingly, the earliness of the ELAN would then reflect an incremental parsing process in which syntactic information of incoming words is checked against a single predicted candidate category (e.g., noun) or its left-side modifiers (e.g., adjectives). Overall, the use of structural information driving categorical expectations converges on data showing that preceding context facilitates different stages of linguistic analysis, including orthographic or phonological processing, lexical access and semantic integration (see Kuperberg and Jaeger, 2016 and Pickering and Gambi, 2018 for two recent reviews).

Evidence for the existence of categorical predictions in language comes from behavioural, neurophysiological, and hemodynamic data. At the behavioural level, predictions driven by syntactic structure have been shown to influence fixation times when reading sentences (Boston et al., 2008). A second eye-tracking study found anticipatory eye-movements suggesting that participants used syntactic structures to inform categorical predictions (Bonhage et al., 2015). At the neurophysiological level, Lau et al. (2006) showed that the amplitude of the ELAN depends on the strength of categorical predictions induced by the previous context. In particular, when an ellipsis configuration softened the prediction for an upcoming noun, an ungrammatical continuation of the sentence (e.g., “*Although Erica kissed Mary’s mother, she did not kiss Dana’s of the bride”, with “of” being the time-locking point for the ERP analysis) led to a reduced negativity compared to when, in a non-ellipsis configuration (e.g., “*Although the bridesmaid kissed Mary, she did not kiss Dana’s of the bride”, with “of” being the time-locking point for the ERP analysis), a noun was strongly expected. This study provided initial evidence for the dependence of the ELAN on categorical predictions, which motivated the use of syntactic violations to investigate top-down linguistic processes in subsequent studies (Dikker et al., 2009, 2010; Jakuszeit et al., 2013). Similarly, evidence for the existence of categorical predictions can be found in studies which do not employ syntactic violations (Matar et al., 2019). Converging evidence comes from electroencephalography (EEG) studies employing narratives, which showed that metrics reflecting grammar-based expectations predict the signal elicited by incoming words (Hale et al., 2018; Brennan and Hale, 2019). Finally, at the two-word level increased oscillatory synchronization has been linked to the anticipatory processes during syntactic composition (Segaert et al., 2018; Hardy et al., 2022).

At the neuroanatomical level, syntactic violations are known to engage the left perisylvian cortex (Vandenberghe et al., 2002; Friederici et al., 2003; Herrmann et al., 2012). Activity in these regions seems to be modulated by syntactic surprisal (Henderson et al., 2016), a metric reflecting how much the current grammatical information is unexpected given the previous context (Hale, 2014). Similar effects have been reported with surprisal indexes estimated according to context-free phrase structure (Brennan et al., 2016) and probabilistic context-free (Shain et al., 2020) grammars as predictors. However, it is unclear whether these studies isolated brain regions involved in generating or checking grammatical predictions. Conversely, the generation of categorical predictions was directly investigated by Bonhage et al. (2015), combining fMRI and eye-tracking methods. In this experiment, the fMRI analysis was constrained by the timing of prediction generation, indicated by the anticipatory eye-movements towards the position of a target category. When only the structural information could be extracted from the context, increased activation as a function of syntactic prediction was observed in Broca’s area. Broca’s area structural (Finkl et al., 2020) and functional (Trettenbrein et al., 2020) profile points towards a role in modality-independent linguistic computations, based on grammar (Chen et al., 2019; Chen et al., 2021a,b). This region is well-known to support linguistic composition, as shown by numerous fMRI studies (Snijders et al., 2009; Tyler et al., 2010; Pallier et al., 2011; Schell et al., 2017; Zaccarella et al., 2017a; van der Burght et al., 2019; Graessner et al., 2021b), lesion data (Friederici et al., 1998, 1999; Graessner et al., 2021a) and meta-analytical findings (Hagoort and Indefrey, 2014; Zaccarella et al., 2017b). Broca’s area pars opercularis (Brodmann area, BA, 44) has been specifically linked to syntactic composition based on abstract categorical representations, as structure-building effects in this region are also observed during the processing of jabberwocky phrases or sentences (Goucha and Friederici, 2015; Zaccarella and Friederici, 2015b). Given that in jabberwocky conditions content elements (e.g., nouns, verbs, adjectives) are replaced by pseudo-words, activity in BA44 might be amplified by the highly predictive nature of the functional elements (determiners, prepositions, morphological particles) retained within the stimuli. Furthermore, increased directed connectivity from BA44 to the posterior left middle temporal gyrus (MTG) is observed when two-word phrases start with a function word compared to a non-predictive element (Wu et al., 2019), possibly reflecting the top-down transmission of a categorical expectation. Interestingly, structural and lexical predictive processes have been linked to Broca’s area activation in a series of studies (Roll et al., 2015, 2017; Söderström et al., 2018). Thus, given the role of Broca’s area in category-based syntactic composition (Friederici, 2011; Hagoort and Indefrey, 2014; Zaccarella et al., 2017b; Grodzinsky et al., 2021), an involvement of this region in categorical predictive processes is a reasonable hypothesis.

This hypothesis about Broca’s area involvement in generating categorical predictions is coherent with computational parsing models (Abney and Johnson, 1991; Resnik, 1992) and functional data from the neuroimaging literature. However, conflicting evidence and theoretical views have also been reported. First, words whose grammatical category is not expected, but which can nonetheless be integrated in a grammatical construction, do not seem to elicit an ELAN (Friederici et al., 1996). This early independence between grammaticality and predictive mechanisms has been also reported in sMMN-based studies, where the neural response to different grammatically correct phrases is not modulated by the frequency of occurrence of the phrase under analysis (Pulvermüller and Assadollahi, 2007; Herrmann et al., 2009). Secondly, given that increased activity in Broca’s area is also observed for syntactic categorical and agreement violations (Carreiras et al., 2010; Heim et al., 2010; Herrmann et al., 2012), it is possible that this brain region may license syntactic structures via a bottom-up process rather than a top-down prediction. As a matter of fact, a recent fMRI study showed that Broca’s area activity correlates with indexes of bottom-up integration during naturalistic listening (Bhattasali et al., 2019). Similarly, increased activity in the left inferior frontal gyrus (IFG) has been reported as a function of whether a word can be integrated in the syntactic context (Hultén et al., 2019). Third, recent data suggest that a careful examination of apparent pre-activation effects is necessary (Nieuwland, 2019). While initial data supported the hypothesis that probabilistic information can be used to anticipate properties of upcoming words up to the phonological level (Delong et al., 2005; Van Berkum et al., 2005), recent large-scale replication studies question this notion (Nieuwland et al., 2018, 2020). Similarly, the modulation of the ELAN by structural predictions originally reported by Lau et al. (2006) was not replicated by a follow-up study (Kaan et al., 2016; see also Nieuwland, 2019 for a discussion of Lau et al., 2006 findings). Finally, theoretical views have emerged which put forward the notion that prediction might not be a necessary component of language comprehension (Huettig, 2015; Huettig and Mani, 2016). Recent behavioural data converge on this notion, showing that syntactic contextual effects at the two-word level might be better described as arising at the integration phase, rather than at the predictive one (Pyatigorskaya et al., 2021).

At present, no causal evidence exists for or against the existence of categorical predictive processes located in Broca’s area. The absence of the ELAN in patients with lesions in Broca’s area (Friederici et al., 1998, 1999) supports a causal role of this region in syntactic composition, but does not discriminate between predictive and bottom-up processes. Both accounts are compatible with the absence of the ELAN, either because no categorical expectation is formed or because the integration phase is disrupted. Here we begin to address the computational role of Broca’s area in syntactic composition by testing one of the two competing hypotheses. In particular, we tested the causal role of Broca’s area in generating categorical predictions by using focal perturbations induced by short trains of Transcranial Magnetic Stimulation (TMS). When delivered “online” (i.e., during the task), TMS allows to test causal relationships between the targeted area and a specific cognitive process of interest (Pascual-Leone et al., 1999; Walsh and Cowey, 2000; Hartwigsen, 2015). Our experiment represents the first investigation of the causal involvement of Broca’s area in generating syntactic predictions by combining three elements:

1. An ESN paradigm in which syntactic categorical predictions can be generated at the basic two-word level (determiner → prediction for a noun, pronoun → prediction for a verb), and fulfilled (grammatical constructions) or violated (ungrammatical constructions).
2. An ERP analysis measuring the different brain responses to prediction fulfilment and violation.
3. A TMS approach with high temporal resolution to causally link Broca’s area to a specific stage of syntactic analysis (i.e., the generation of predictions).

If Broca’s area is causally involved in syntactic predictive processes, TMS-induced disruption of this region during the prediction phase should attenuate the difference between expected and unexpected categories. Specifically, we expect to find a Grammaticality × TMS interaction on the ESN amplitude. Conversely, if our data do not provide evidence for a causal role of Broca’s area in syntactic prediction, two alternative hypotheses can be formulated. According to the first hypothesis, additional nodes of the syntactic network might have compensated the transient and short-lived disruption of Broca’s area induced by TMS (Hartwigsen, 2018, see also Hartwigsen et al., 2016 and Kroczek et al., 2019 for a discussion of similar effects in the semantic domain). According to the second alternative hypothesis, a bottom-up role of Broca’s area in incremental parsing can be proposed, namely the integration of syntactic units into constituents. This hypothesis is in line with the involvement of the left inferior frontal gyrus (IFG) in bottom-up parsing (Bhattasali et al., 2019) and in syntactic violation detection (Heim et al., 2010; Carreiras et al., 2012; Herrmann et al., 2012). Moreover, at a broader theoretical level, this hypothesis converges on theoretical views questioning the role of prediction in language comprehension (Huettig, 2015; Huettig and Mani, 2016).

## Materials and methods

### Participants

A total of 30 native German speakers were recruited for the experiment. Due to the presence of strong artifacts in the EEG signal, one subject was excluded from the analysis. Therefore, 29 subjects were included in the statistical analysis (15 females, 14 males; mean age: 27.1 years, standard deviation: 4.1 years). All participants were right-handed (mean laterality quotient: 93.3, standard deviation: 9.5), as assessed with the Edinburgh handedness test (Oldfield, 1971), had normal or corrected-to-normal vision, and no colour blindness. None of the participants presented contraindications against TMS or had history of psychiatric or neurological disorders. Participants gave their written informed consent and were reimbursed 12€ per hour for participating in the study. The study was approved by the local ethics committee (University of Leipzig) and was conducted in compliance with the Declaration of Helsinki guidelines.

### Paradigm

Our experiment employed an adapted version of a standard two-word auditory ESN paradigm with syntactic categorical violations (Hasting et al., 2007; Hasting and Kotz, 2008; Herrmann et al., 2009, 2012; Jakuszeit et al., 2013). The first word of each utterance was the German determiner “Ein” (*a*) or the personal pronoun “Er” (*he*), while the second word could be either a noun or verb. A total of 32 pairs of nouns and verbs with an ambiguous stem were used (e.g., “Fal-ter”, *butterfly*, and “Fal-ter”, *folds,* see “Stimuli” section). Each second word was presented once following the determiner and once following the personal pronoun, resulting in four possible types of trials, two grammatical (*a* + *noun*, *he + verb*) and two ungrammatical (*a + *verb*, *he + noun). The grammatical and ungrammatical conditions consisted of 64 trials each, as 32 pairs of nouns and verbs were used. The conditions of the paradigm are summarised in Table 1. Grammaticality constitutes the first factor in our experimental design, reflecting whether the second word matched the categorical prediction triggered by the first one (grammatical items) or not (ungrammatical items). Importantly, with this paradigm grammaticality is orthogonal to both the identity of the first word (“Ein” or “Er”) and the grammatical category of the second word (noun or verb), therefore ruling out potential methodological issues discussed in the context of ELAN studies (Steinhauer and Drury, 2012). For example, it has been pointed out that pre-target differences between grammatical (e.g., “wurde gegessen”, *was eaten,* with “ge” serving as time-locking point) and ungrammatical (e.g., “*wurde im gegessen”, *was in-the eaten, with the prefix “ge-” serving as time-locking point) structures might have contributed to the ELAN effects reported in the literature (Steinhauer and Drury, 2012). Compared to the ELAN studies, the ESN paradigm offers the advantage that the pre-target information is matched across conditions (see also “Stimuli” section). Furthermore, the ESN is time-locked to the point of category access of the second word (see “EEG recording and analysis” section). This overcomes potential limitations of some ELAN studies, such as the time-locking to a point where linguistic information (e.g., “ge-”) is indicative of a grammatical category (e.g., paste participle) but not uniquely associated to it (Steinhauer and Drury, 2012). As previously shown (Hasting and Kotz, 2008), ungrammatical items result in an increased ESN response, functionally equivalent to the ELAN observed with longer stimuli (Friederici, 2011). In a follow-up study, Jakuszeit et al. (2013) reported an increased late positivity, functionally equivalent to the P600 component, for ungrammatical conditions.

**Table 1:** Conditions included in the experimental paradigm.

Experimental conditions	
**Grammatical**	**Ungrammatical**
Ein Falter (*a butterfly*) Er faltet (*he folds*)	*Ein faltet (*a folds) *Er Falter (*he butterfly)

The design crossed Grammaticality (grammatical vs. ungrammatical) and TMS (Brodmann Area (BA) 44, superior parietal lobe (SPL) and sham).

### Stimuli

As in the original version of the paradigm (Hasting and Kotz, 2008), we used nouns and verbs with ambiguous stems in which the categorical information can only be accessed once the suffix is processed (e.g., “Fal-ter”, *butterfly*, and “fal-tet”, *folds*) as second words. In this way, we could precisely time-lock the ERP analysis to the point of categorical access of the second word, which is represented by the suffix onset time. While in the original study (Hasting and Kotz, 2008) the category of most of the nouns was expressed with zero marking (e.g., “Kegel-Ø^2^”, *cone*, compared to the verb “kegel-t”, *bowls*), we decided to include only nouns with the category overtly marked by a suffix. Our decision was motivated by studies which showed no syntactic categorical violation effects when nouns with zero marking were used (Dikker et al., 2009; Herrmann et al., 2009). Furthermore, syntactic violations realised with an offending suffix (e.g., “*Er kegel-st”, *he bowl) are more robust against conditions of reduced statistical power such as small or heterogeneous sample sizes than unmarked ones (e.g., “*Er Kegel- Ø”, *he cone, Jakuszeit et al., 2013).

The 32 pairs of nouns and verbs with ambiguous stems used in our experiment are the result of a four-step selection procedure (see also the Appendix for the full list of nouns and verbs used). First, we extracted from CELEX corpus (Baayen et al., 1995) masculine and neuter German disyllabic nouns ending in “-er”. We used only masculine and neuter nouns in the nominative case as they follow the determiner “Ein”, with limited agreement processes involved. Secondly, for each noun we constructed a potential verb “candidate” in the infinitive form, by replacing the suffix “-er” with the infinite form ending “-en” (e.g., “Falt-er”, *butterfly* → “falt-en”, *to fold*). For nouns ending with “-ler”, we constructed an additional infinite candidate form using the ending “-eln” (e.g., “Schwin-dler”, *cheater* → “schwin-deln”, *to cheat*). Nouns for which the respective verb candidate was not found in CELEX corpus were excluded at this step. In the third step, the verbs were inflected in the present tense third-person singular form. Pairs in which the verb became monosyllabic when inflected were excluded from the list. Finally, as in German “Ein” and “Er” can form compounds prefixing both nouns and verbs, we removed pairs of nouns and verbs in which the ungrammatical forms could exist as a compound (i.e., “ein + verb” or “er + noun”) according to the majority of eight independent native German speakers. The auditory stimuli used in the experiment were prepared adapting the cross-splicing procedure described by Hasting and Kotz (2008). For each pair of nouns and verbs a trained German native speaker was asked to read several times three utterances:

- The correct determiner phrase (e.g., “Ein Fal-ter”, *A butterfly*);
- The correct pronoun and verb construction (e.g., “Er fal-tet”, *he folds*);
- The stem embedded in a meaningless pseudo-word phrase (e.g., “Lub fal-tek”).

The recordings were acquired in a soundproof cabin using Audacity software (sampling rate: 44100 Hz). The most similar determiner phrase, pronoun and verb construction and pseudo-word phrase were then selected for the cross-splicing procedure. From the determiner phrase, the word “Ein” and the noun suffix (e.g., “-ter”) were extracted. The pronoun “Er” and the verb suffix (e.g., “-tet”) were then extracted from the pronoun and verb construction. When the two first words (“Ein” and “Er”) were extracted from the recordings, also the silence extending up to 600 ms (the closest zero-crossing sample) from word onset was included. Similarly, the stem (e.g., “Fal”) was extracted from the pseudo-word phrase. To avoid clicking sounds, the recordings were cut only at points of zero crossing. The grammatical and ungrammatical utterances (e.g., |Ein|Fal|-ter|, ^*^|Ein|Fal|-tet|, |Er|Fal|-tet|, *|Er|Fal|-ter|) were then created by concatenating one of the two first words (e.g., |Ein| or |Er|), the stem (e.g., |Fal|), and one of the two possible suffixes (e.g., |-ter|, |-tet|). Finally, the constructed utterances were normalized to 65 dB and 7 ms of silence were added at the beginning of each stimulus. Since TMS pulses produce a loud click noise, concatenated items were normalized to adjust the volume of the stimuli at the beginning of the experiment so that all the utterances could be heard clearly. Manipulation of the recordings was performed using Praat software (Boersma, 2001). Our procedure strongly reduced acoustic differences between grammatical and ungrammatical utterances up to the divergence point (DVP), after which the suffix occurs and the category of the second word is revealed (e.g., Ein Fal_[DVP]_ter, *Ein fal_[DVP]_tet, Er fal_[DVP]_tet, *Er Fal_[DVP]_ter). A t-test on the root mean square amplitude of the recordings up to the DVP revealed no significant difference between grammatical (Ein + Noun, Er + Verb) and ungrammatical (Ein + Verb, Er + Noun) items (*p = 0*.99).

### Transcranial magnetic stimulation

Transcranial magnetic stimulation was applied during the task (“online”) to investigate the causal role of BA44 in syntactic predictive processes. We delivered 10 Hz trains of five TMS pulses during the first word of each item (“Ein”, *a*, or “Er”, *he*) to perturb the stage of syntactic categorical prediction (determiner → prediction for a noun, pronoun → prediction for a verb). The first pulse of each TMS train was time-locked to the onset of the first word and each burst lasted 400 ms. Since the second word of each item started 600 ms after the first word onset and potential after-effects of online TMS are thought to last approximately half of the stimulation time (Rotenberg et al., 2014), our stimulation protocol and stimuli materials ensured that the perturbation was limited to the stage of syntactic prediction only.

**Figure 1:**
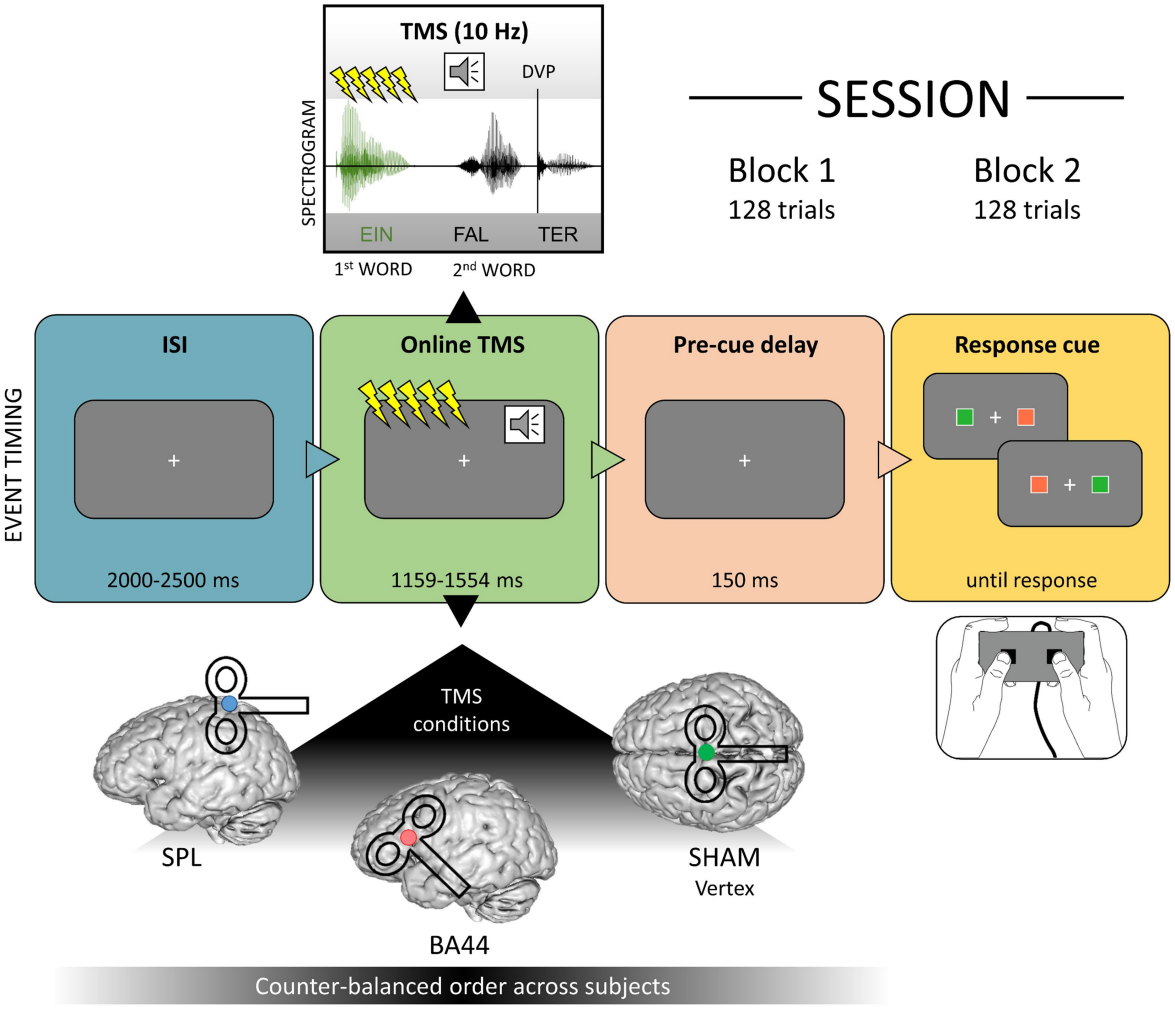
Timing of events including an illustration of the online stimulation during the first word (above the event timeline) and the three target regions for the neuronavigation system (below the event timeline). Divergence Point (DVP); Interstimulus Interval (ISI); Superior parietal lobe (SPL); Brodmann Area (BA) 44.

We included three TMS conditions: BA44, the left superior parietal lobe as an active control site, and a sham condition (Figure 1). Each participant took part in three experimental sessions, one for each TMS condition, which were separated by at least 7 days (mean distance: 7.89 days, standard deviation: 2.96 days). The order of conditions was counter-balanced across subjects. The precise cortical stimulation of the target regions was ensured by employing a neuronavigation system (TMS Navigator software version 3.0.33, Localite GmbH, Sankt Augustin, Germany), which calculated for each participant the optimal positions of the TMS coil on the scalp based on MNI coordinates. The MNI coordinates for BA44 (MNI: −48, 17, 16) were defined according to the results of Zaccarella and Friederici (2015b), who found increased activation for phrases compared to word lists in this region. This target is located in the most anterior and ventral part of BA44, which is suggested to be functionally specialized in syntactic computations (Papitto et al., 2020; Zaccarella et al., 2021). The SPL coordinates (SPL, MNI: −34, −42, 70) were based on a TMS experiment on degraded speech comprehension in which this region served as a control condition (Hartwigsen et al., 2015). Note that, despite the fact that a single voxel serves as a virtual target for the neuronavigation system, the effect of TMS is usually characterized by a spread in its vicinity. As discussed in the “ERP and induced electrical field simulation” section, our analysis employed state-of-the-art procedures (Weise et al., 2020) to precisely model this aspect of TMS-induced electrical fields. In the sham condition, no effective stimulation of the brain occurred. The vertex was chosen as a target for sham TMS to perform the neuronavigation procedure as in the other TMS conditions (Klaus and Hartwigsen, 2019; Friehs et al., 2020; Kuhnke et al., 2020). In the sham condition, a disconnected coil was navigated over the electrode Cz and an active coil was placed above it with an angle of 90°, therefore not stimulating the brain. This procedure produces the same acoustic noise as the other two TMS conditions, without an actual stimulation of the brain (Kuhnke et al., 2017, 2020; Meyer et al., 2018; Kroczek et al., 2019; Friehs et al., 2020).

We used stereotactic neuronavigation (TMS Navigator software version 3.0.33, Localite GmbH, Sankt Augustin, Germany) to position and maintain the TMS coil over the target regions during the experiment. Individual structural T1-weighted MRI images were previously acquired for each participant. The coordinates of the target regions were converted from the MNI standard to the individual subject space using SPM12 software (Wellcome TrustCenter for Neuroimaging, University College London, United Kingdom), using an established procedure (Kuhnke et al., 2017, 2020; Klaus and Hartwigsen, 2019; Friehs et al., 2020). After EEG preparation, the head of each participant was co-registered to their MRI image, allowing for precise positioning of the TMS coil over the target coordinates as defined in the individual anatomical image.

TMS was delivered using a figure-of-eight coil (C-B60) connected to a MagVenture MagPro X100 stimulator (MagVenture, Farum, Denmark). The coil handle was oriented with an angle of 45° and 0° relative to the sagittal plane when stimulating BA44 and the SPL respectively, as in previous studies (Hartwigsen et al., 2015; Kuhnke et al., 2017; Meyer et al., 2018; Klaus and Hartwigsen, 2019; Kroczek et al., 2019). The intensity of the stimulation was set to 90% of the individual resting motor threshold (RMT), defined during the first session for each participant. RMT was defined as the minimum intensity at which TMS could evoke at least 5 motor evoked potentials (MEP) with an amplitude ≥50 μV in the relaxed first dorsal interosseous muscle out of 10 consecutive pulses (Rothwell et al., 1999). To this end, the TMS coil was navigated over the coordinates of the left hand motor area (MNI: −37, −21, 58, Mayka et al., 2006) and the hotspot was identified with a standard threshold hunting procedure. If necessary, stimulation intensity for BA44 was corrected for the scalp-to-cortex distance relative to the motor cortex. The adjusted intensity was calculated using the formula by Stokes et al. (2005), as adapted for applications with 90% of the RMT (Kuhnke et al., 2017): BA44 intensity (stimulator output) = 90% RMT + 3*(Distance_BA44_ – Distance_M1_), where Distance_BA44_ and Distance_M1_ correspond to the distance in mm between the scalp and BA44 and M1, respectively. The stimulation intensity for the sham condition was the same as the one used for BA44. Finally, the stimulation intensity for the SPL condition corresponded to the 90% of the RMT, as for no subject it required to be adjusted. If stimulation was too unpleasant, intensity was gradually decreased in steps of 1%.

### Procedure and timing of events

At the beginning of the TMS-EEG sessions, each participant filled in a short TMS safety questionnaire and received the task instructions. After EEG preparation, the participant was moved into an electrically shielded cabin where the head surface was co-registered to the structural MRI image for TMS neuronavigation. Subjects sat comfortably approximately 140 cm from the computer monitor. During the first TMS-EEG session, the individual RMT was defined. To familiarize the subject with the sensory stimulation associated with each TMS condition, some test pulses on the target region were delivered. Before the experiment, subjects were provided with in-ear headphones and after reading a reminder of the instructions they underwent a short practice block, consisting of 12 trials with items excluded from the main task. The trial structure of the practice block was the same as the one of the main task, but feedback was provided after each response to ensure that subjects understood the instructions. To provide comparable conditions, TMS was also delivered during the practice trials, allowing the subject to indicate if the sound volume needed to be adjusted due to the TMS-induced noise.

During the task, the TMS coil was manually positioned and maintained over the target region. Subjects performed a grammaticality judgement task, indicating if the two-word utterance they heard was grammatically correct or not via a button-box press. A fixation cross was displayed at the centre of the monitor, and after an inter-stimulus interval randomly jittered between 2 and 2.5 seconds (s) the two-word item was presented acoustically. The TMS train was delivered during the first word. After the acoustic item ended, a delay of 150 ms was included to avoid an overlap of language-related and motor-related evoked responses in the EEG signal. A response cue was then presented, consisting of two coloured squares presented to the left and right of the fixation cross. One of the squares was red and one green, with the colours being assigned pseudo-randomly for each trial. The green colour was associated to the position of the response button for “grammatical”, similarly the red colour coded for “ungrammatical”. We used a red and a green colour with a similar luminance (L = 64.39 and 64.37, respectively, in CieLuv color-space) to avoid that differences in brightness might bias the behavioural data analysis. Relative luminance was calculated implementing the formula defined in the Web Content Accessibility Guidelines (WCAG) 2.0 (https://www.w3.org/TR/WCAG20/Overview.html#Srgb, see Supplementary Materials). The timing of TMS bursts and stimulus presentation was controlled using Presentation Software version 17.2 (NeurobehavioralSystems, Inc., Albany, CA, United States). Figure 1 illustrates the structure of a trial. In each session, subjects performed the task twice (in two blocks), with the same 128 items presented in a different pseudo-randomized order. Short breaks were included every 32 trials, to cool down and switch the TMS coil if needed. After the experiment, the position of the electrodes was digitized using the TMS Navigator software. Considering TMS-EEG preparation, the first experimental session lasted on average approximately 3.5 h, while the other two lasted approximately 2.5 h.

### Behavioural data analysis

Behavioural data were analysed with repeated measures analysis of variance (ANOVA), including as factors Grammaticality (grammatical and ungrammatical), TMS (BA44, SPL and sham) and Block (first and second). Repeated measures ANOVAs were conducted in R (R Core Team, 2020) on the subjects’ mean responses times and accuracy rates for each condition, following the removal of trials with RTs shorter than 150 ms or longer than 1 s. Analysis of the RTs was based on trials with correct response only. The behavioural data analysis was conducted in R (R Core Team, 2020) using the package “ez” (Lawrence, 2016).

#### EEG recording and analysis

TMS pulses result in a series of artifacts on the concurrent EEG signal which need to be controlled during both data collection and pre-processing. Electromagnetic artifacts are commonly observed following each TMS pulse (Veniero et al., 2009; Ilmoniemi and Kičić, 2010; Rogasch et al., 2013, 2014), and depending on the target location additional large cranial muscular activity can contaminate the EEG signal. Muscle artifacts are particularly pronounced when the target site is a lateral brain region (Mutanen et al., 2013; Rogasch et al., 2013), such as the IFG and the posterior temporal lobe (Salo et al., 2020). Given the series of potential artifacts during online TMS-EEG, the employed procedure for data collection and pre-processing in the present study differs from traditional EEG studies.

EEG signal was recorded using 63 Ag/AgCl monopolar electrodes (61 electrodes embedded in an EEG cap, EC80, EasyCap GmbH, Germany, and A1 and A2 on the left and right mastoids respectively), which were placed according to the international extended 10–20 system. Two additional pairs of bipolar electrodes were placed to monitor vertical and horizontal eye movements. EEG signal was amplified using REFA8 68-channel amplifier system (TMSi, Oldenzaal, the Netherlands) and recorded at a sampling rate of 2000 Hz using BrainVision Recorder software version 1.02.0001 (Brain Products GmbH, Gilching, Germany). The average of the 63 monopolar electrodes served as an online reference. The ground electrode was placed on the sternum. Electromagnetic artifacts following each TMS pulse were reduced by arranging the direction of the electrode wires orthogonally to the TMS coil handle (Sekiguchi et al., 2011). Impedance was kept below 5 kΩ.

Pre-processing was performed using the Matlab FieldTrip toolbox version fieldtrip-20200115 (Oostenveld et al., 2011). Given that the TMS trains were time-locked to the first word onset, EEG signal in this time-window was strongly contaminated by the large electromagnetic and muscular artifacts described at the beginning of this section. The presence of these artifacts could have resulted in large signal distortions when applying common EEG pre-processing steps like filtering on the raw data (Rogasch et al., 2017). To overcome this issue, cubic interpolation of the signal contaminated by TMS artifacts is usually employed (e.g., Rogasch et al., 2013; Herring et al., 2015; Kroczek et al., 2019), allowing a smooth transition with the remaining EEG signal.^3^ Since our ERP component of interest is time-locked to the DVP of the second word, we applied cubic interpolation of the continuous EEG signal from −2 to 450 ms relative to the first pulse of each TMS train (first word onset). Cubic interpolation was based on the 300 ms time-window before and after the segments to be interpolated. The continuous EEG signal obtained after interpolation was high-pass filtered with a cutoff frequency of 0.5 Hz (onepass-zerophase, order 4460, kaiser-windowed sinc FIR, 6 dB attenuation at the cutoff frequency, transition width 1.0 Hz, stopband 0–0.0 Hz, passband 1.0–1,000 Hz, max passband deviation 0.0100, stopband attenuation 40 dB). This cutoff frequency was chosen in order to match the low cutoff frequency in the bandpass filter used by Jakuszeit et al. (2013), which to the best of our knowledge is the last follow-up study employing the original ESN paradigm. Epochs from −250 ms to 2 s relative to the DVP of the second word were then extracted. Epochs were visually inspected and trials and channels with excessive artifacts were removed (trials removed per block: mean = 3.4, standard deviation = 3.5; channels removed per block: mean = 0.9, standard deviation = 0.9). The common average reference of the good channels was then computed, and Independent Component Analysis (ICA) using the RunICA algorithm was run, accounting for data rank reduction due to bad channel exclusion. ICA components were visually inspected and bad components reflecting ocular, cardiac and muscle artifacts were removed. If present, components reflecting the exponential decay after TMS were removed as well. After the removal of bad ICA components (number of components kept per block: mean = 27.1, standard deviation = 5.2), EEG data were re-referenced to the common average and the signal of the channels removed during visual inspection was interpolated using spherical spline interpolation (Perrin et al., 1989), an approach recently used in a TMS-EEG experiment targeting Broca’s area (Kroczek et al., 2019). EEG data were then re-referenced to the new common average reference and trials with an incorrect response were removed. The clean trials with a correct response were low-pass filtered with a cut-off frequency of 44 Hz (onepass-zerophase, order 408, kaiser-windowed sinc FIR, 6 dB attenuation at the cutoff frequency, transition width 11.0 Hz, passband 0–38.5 Hz, stopband 49.5–1,000 Hz, max passband deviation 0.0100, stopband attenuation 40 dB). These pre-processing steps were repeated for each of the two blocks in each session. The trials from the two blocks were then merged in one unique dataset per TMS condition for each subject and re-referenced to the average of A1 and A2 electrodes. No baseline correction was applied, as the use of our high-pass filter already attenuated direct-current offset (Widmann et al., 2015). From each dataset two ERP waveforms were then calculated, averaging separately the trials belonging to the grammatical and ungrammatical conditions. This procedure resulted in six ERP waveforms per subject, reflecting the six cells of our Grammaticality × TMS within-subject design. ERP waveforms were then calculated to test three effects of interest: the main effect of Grammaticality (averaged across TMS conditions), the main effect of TMS (averaged across stimulus conditions in each session) and the interaction between Grammaticality and TMS. Each ERP waveform was calculated averaging approximately 120 trials. The average numbers of trials entering each ERP averaged waveform, together with the respective standard deviation (between brackets), were the following: 120.48 (6.23) for BA44 grammatical, 119.58 (5.61) for BA44 ungrammatical, 121.41 (3.05) for SPL grammatical, 121.31 (4.13) for SPL ungrammatical, 120.76 (5.67) for sham grammatical, 120.344 (5.92) for sham ungrammatical.

The statistical analysis of EEG data was performed using non-parametric cluster-based permutation tests (Maris and Oostenveld, 2007) implemented in the FieldTrip toolbox (Oostenveld et al., 2011). The dependent sample T-statistic (“depsamplesT”) was used for cluster formation when analysing the main effect of Grammaticality. For the analysis of the main effect of TMS and the Grammaticality × TMS interaction, the dependent sample F-statistic (“depsamplesFunivariate”) was used, as three levels were present in the independent variable.^4^ The cluster-level statistic was calculated as the maximum of the cluster-level summed *T*- or *F*-values of each cluster. The critical alpha level for the Monte Carlo significance probability was set to 0.025 when testing the main effect of Grammaticality (two-tailed hypothesis) and to 0.05 for the analysis of the main effect of TMS and the Grammaticality × TMS interaction (one-tailed hypothesis). In each of the three statistical tests conducted, the Montecarlo estimation was based on 5,000 random partitions and the time-window of interest was defined from 0 to 1,000 ms relative to the DVP.

#### Bayesian repeated measures ANOVA on the ESN

To quantify the evidence for and against the presence of a Grammaticality × TMS interaction in our EEG data, we performed an additional Bayesian repeated measures ANOVA on the mean amplitude of the ESN. Bayesian analysis allows to quantify evidence for both the null and the alternative hypotheses, describing how informative data from a given experiment are (Wagenmakers et al., 2018b; Keysers et al., 2020). Bayes factors (BF) indicate how likely the data are under these two hypotheses. For example, a BF_10_ equal to 5 indicates that the current data are five times more likely under the alternative than the null hypothesis. BF_01_ is equal to 1/BF_10_ and indicates how many times the data are more likely under the null hypothesis. In a Bayesian repeated measures ANOVA, Bayes Factors are obtained by comparing the predictive performance of two models (Wagenmakers et al., 2018a; van den Bergh et al., 2020). Bayes Factors BF_10_ and BF_01_ quantify how much the data are more likely according to one of the two competing models (e.g., an alternative model against the null model or the best model).

The analysis was conducted using JASP software version 0.14 (JASP Team, 2020 ^5^; for theoretical and practical introductions see Wagenmakers et al., 2018a,b; van Doorn et al., 2021; Faulkenberry et al., 2020; Keysers et al., 2020). The Bayesian repeated measures ANOVA included Grammaticality and TMS as factors. This analysis compared the performance of five models: a null model (M0: coding only the presence of different subjects) and four alternative models (M1: subject + Grammaticality, M2; subject + TMS, M3: subject + Grammaticality + TMS, M4: subject + Grammaticality + TMS + Grammaticality × TMS). The default uninformed prior distribution was used. We planned to test the Grammaticality × TMS interaction in two ways:

1. By comparing model M4 including the interaction against the models which included only the main effect of Grammaticality (M1) and the two main effects (M3). This comparison quantifies how much adding an interaction term improves the predictive performance of the model.
2. By performing an analysis of the effects via Bayesian Model Averaging (Wagenmakers et al., 2018a; Hinne et al., 2020; Keysers et al., 2020; van den Bergh et al., 2020). With this analysis BF_incl_ and BF_excl_ are obtained, indicating respectively how much more likely the data are under models which include and exclude a given factor or interaction. The analysis of effects was computed across all models.

For the Bayesian repeated measures ANOVA on the ESN, we extracted the mean amplitude of the waveforms averaging signal between 190 ms and 430 ms at 41 electrodes: AF3, AFz, AF4, F5, F3, F1, Fz, F2, F4, F6, FC5, FC3, FC1, FCz, FC2, FC4, FC6, C5, C3, C1, Cz, C2, C4, C6, CP5, CP3, CP1, CPz, CP2, CP4, CP6, P5, P3, P1, Pz, P2, P4, P6, PO3, POz, and PO4. The electrodes and time-points included are based on the results of the main effect of Grammaticality (negative cluster) and by the rather spread topography of our ERP component of interest (see section “EEG data” below). Henceforth we refer to this as the Full ESN. Crucially, the criterion used for selecting the electrodes and time-points included does not make circular the analysis, which addresses a different research question (interaction) compared the test used for defining them (main effect of Grammaticality).

A similar analysis, conducted on the late positivity/P600, is reported in the Supplementary Tables S1-S3.

#### ERP and induced electrical field simulation

Together with stimulation intensity and coil orientation (Laakso et al., 2014; Weise et al., 2020), neuroanatomical factors such as individual gyrification patterns (Thielscher et al., 2011) and the distribution of tissue types (Opitz et al., 2011; Lee et al., 2018) affect the spread and strength of the electrical field induced by TMS pulses. To precisely characterize the impact of BA44 stimulation on the amplitude of the ESN, we performed an additional analysis on the EEG data including the strength of the electrical field in this target region for each subject. By modelling the extent to which TMS interfered with the target region it is possible to account for anatomical factors (Thielscher et al., 2011; Lee et al., 2018) which, differing between subjects, might otherwise hide the presence of an effect of TMS if not included in the analysis (Kuhnke et al., 2020).

The calculation of the induced electrical fields was implemented using a recently established pipeline (Weise et al., 2020). For each subject and each active TMS condition we performed an electrical field simulation based on individual T1-weighted images, additional T2-weighted images if available, and the coil position recorded during the experimental session. Individual head meshes were constructed using the headreco pipeline (Nielsen et al., 2018) and Simnibs software (Windhoff et al., 2013) was used to calculate the electric fields. The electric field models were visually inspected to ensure good quality of the head models. At this stage, two subjects were excluded from the analysis, due to an unrealistic field reconstruction. For each of the remaining 27 subject we extracted the average electrical field intensity from nine regions of interest (ROIs), two in Broca’s area (BA44 and BA45; Amunts et al., 1999, 2004), and seven in the SPL (BA5L, BA5M, BA5Ci, BA7A, BA7PC, BA7M, BA7P; Scheperjans et al., 2008a,b) using maximum probability maps from the SPM Anatomy Toolbox version 2.2c (Eickhoff et al., 2005, 2006, 2007). The inclusion of BA45 as a ROI is motivated by its involvement, together with BA44, in categorical prediction (Bonhage et al., 2015) and by its close proximity to this region in the left IFG.^6^ The average electrical fields in Broca’s area (BA44 and BA45) and in SPL (BA5L, BA5M, BA5Ci, BA7A, BA7PC, BA7M, BA7P) ROIs were extracted from the BA44 and SPL TMS sessions, respectively.

To test whether TMS affected the ESN, we computed a Pearson correlation between the induced electrical field in the abovementioned ROIs and the sham-normalized amplitude of Full ESN. The two sham-normalized Full ESN amplitudes were obtained in a two-step procedure:

1. First, for all the three TMS conditions we calculated the mean amplitude of the difference wave (ungrammatical – grammatical), resulting in three mean amplitude values: Full ESN_BA44_, Full ESN_SPL_ and Full ESN_sham_.
2. We then obtained the sham-normalized mean amplitudes (Full ESN_BA44_ effect, Full ESN_SPL_ effect) by subtracting Full ESN_sham_ from Full ESN_BA44_ and Full ESN_SPL_, respectively (e.g., Full ESN_BA44_ effect = Full ESN_BA44_ – Full ESN_sham_). As the induced electrical field for the sham condition is zero (no electrical stimulation of the brain), this subtraction isolated the effect of the induced field in a given ROI on the ESN amplitude for each of the two active TMS conditions.

Additionally, as the ESN was characterized by an early frontal component and a second centro-parietal component (see “Results” section and Figure 3), we performed an exploratory analysis focusing on each component separately. This additional analysis is motivated by ERP studies showing the presence of two subsequent negativities for agreement (Pulvermüller and Shtyrov, 2003; Barber and Carreiras, 2005; Jakuszeit et al., 2013; Hanna et al., 2014) and categorical (Hasting et al., 2007) marked syntactic violations at the two-word level. We subdivided the Full ESN in two parts:

**Figure 2:**
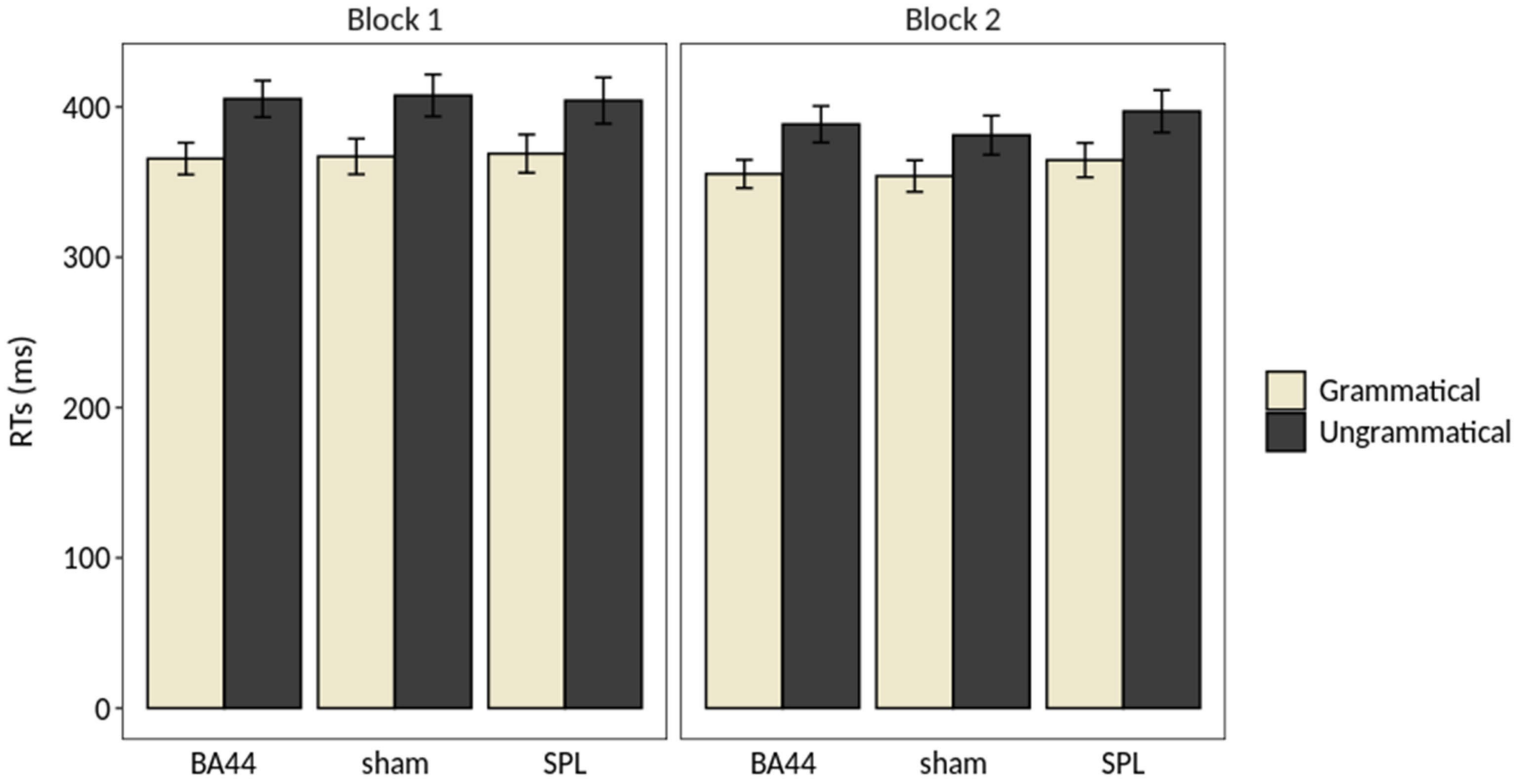
Results of the repeated measures ANOVA on the Response Times (RTs). The error bar indicates the standard error of the mean.

**Figure 3:**
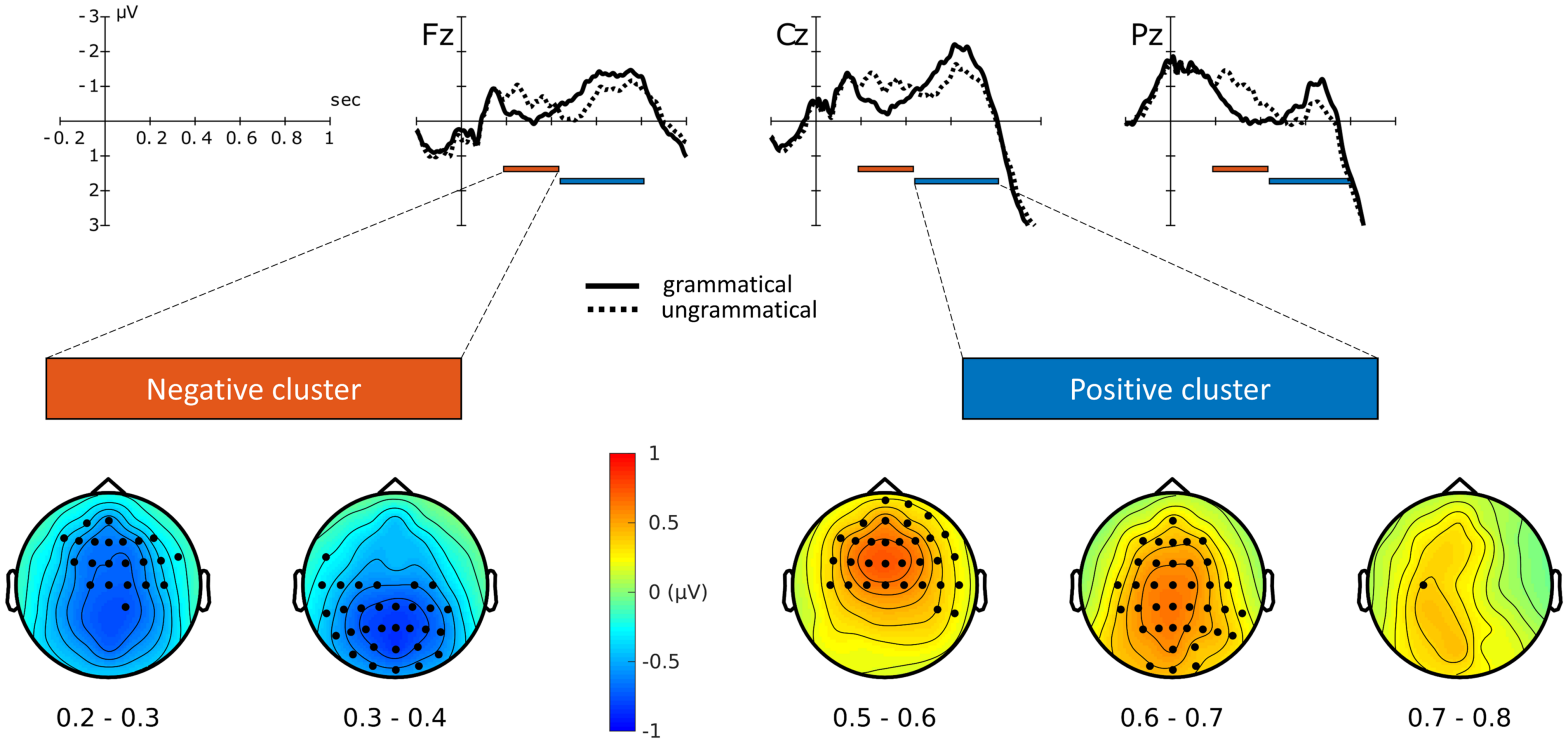
ERP waveforms for grammatical and ungrammatical conditions (µV over seconds, collapsed across TMS conditions at selected electrodes), together with electrodes and time-points providing the highest contribution to the significant clusters.

1. First ESN: average of signal from 190 ms to 310 ms at 17 anterior electrodes AF3, AFz, AF4, F5, F3, F1, Fz, F2, F4, F6, FC5, FC3, FC1, FCz, FC2, FC4 and FC6. The time-points included correspond to the first half of the Full ESN effect. Only anterior electrodes are included, in light of the topography of the main effect of grammaticality in this time-window (see Figure 3).
2. Second ESN: average of signal from 310 ms to 430 ms at 17 posterior electrodes CP5, CP3, CP1, CPz, CP2, CP4, CP6, P5, P3, P1, Pz, P2, P4, P6, PO3, POz and PO4 (second half of the time-window of the Full ESN effect). Only posterior electrodes are included, since in this time-window the effect is mostly pronounced at these sites (see Figure 3).

First ESN_BA44_ effect, First ESN_SPL_ effect, Second ESN_BA44_ effect and Second ESN_SPL_ effect were obtained with the same procedure described above for the full time-window, normalizing First ESN_BA44/SPL_ and Second ESN _BA44/SPL_ with the subtraction of First ESN_sham_ and Second ESN_sham_, respectively.

The NHST correlational analysis was complemented by Bayesian inference using JASP software (JASP Team, 2020), to quantify both evidence for the alternative and the null hypotheses. The default uninformed prior distribution was used.

Similar analyses were conducted for the P600 component. In particular, three P600 effects were correlated with the TMS-induced electrical field in our target regions: Full P600 effect (Full P600_BA44_ – Full P600_sham_), First P600 effect (First P600_BA44_ – First P600_sham_), and Second P600 effect (Second P600_BA44_ – Second P600_sham_). The procedure, mirroring the one implemented for the ESN, is described in detail in the Supplementary Materials.

## Results

### Behavioural data

The performance of the participants was at ceiling (mean accuracy = 97%, range = 75–100%), and the analysis of the accuracy revealed no significant main effect or interaction involving the factors Grammaticality, TMS and Block (Table 2). The analysis of response times (RTs) showed a main effect of Grammaticality (*F*(1,28) = 92.43, *p* < 5e-10, *η*^2^*_G_* = 0.0655), with RTs for the grammatical items being on average 35 ms faster than for the ungrammatical ones. The main effect of Block was significant (*F*(1,28) = 11.35, *p* < 0.005, *η*^2^*_G_* = 0.0097), with RTs being on average 13 ms faster in the second block. Finally, the interaction Grammaticality × Block was significant (*F*(1,28) = 7.20, *p* < 0.05, *η*^2^*_G_* = 0.0008). A *post-hoc* analysis revealed that this interaction was driven by a significant difference between the RTs for the ungrammatical conditions of Block 1 and Block 2 (*p* = 0.001, Bonferroni-corrected), which was absent for the grammatical counterpart (*p* > 0.1, Bonferroni-corrected). No main effect of TMS and no interaction including this factor was significant. Figure 2 illustrates the results of the repeated measures ANOVA on the RTs. Table 2 summarizes the results of the repeated measures ANOVAs on RTs and accuracy rates.^7^

**Table 2:** Results of the repeated measures ANOVAs performed on the RTs and accuracy rates.

			RTs			Accuracy		
Effect	DF_N_	DF_D_	*F*-value	value of *p*	*η* ^2^ *_G_*	*F*-value	value of *p*	*η* ^2^ *_G_*
Grammaticality (Gram)	1	28	92.427	0.000	0.065	1.729	0.199	–
TMS	2	56	0.471	0.627	–	0.123	0.807§	–
Block	1	28	11.355	0.002	0.010	0.742	0.396	–
Gram × TMS	2	56	0.234	0.792	–	0.182	0.834	–
Gram × Block	1	28	7.203	0.012	0.001	0.521	0.476	–
TMS × Block	2	56	1.410	0.253	–	0.125	0.883	–
Gram × TMS × Block	2	56	0.688	0.506	–	0.735	0.484	–

The symbol § denotes a value of *p* adjusted employing the Greenhouse–Geisser procedure (epsilon value: 0.691).

### EEG data

The ERP waveforms of grammatical and ungrammatical conditions at selected electrodes, collapsed across TMS sites, are shown in Figure 3. Additional electrodes are displayed in the Supplementary Figure S1. Visual inspection of the ERP waveforms reveals an increased negativity for the ungrammatical condition from approximately 200 ms to 450 ms, followed by a late positivity from 450 to 800 ms. The cluster-based permutation test revealed a main effect of Grammaticality, with the presence of two significant clusters (*P* < 0.0005, cluster-corrected), one negative and one positive. The negative cluster, reflecting increased negativity for the ungrammatical condition relative to the grammatical one, extended approximately from 190 to 430 ms after the DVP^8^ (Figure 3). The positive cluster, reflecting an effect in the opposite direction, extended approximately from 440 to 800 ms after the DVP. Both the effects were mostly pronounced over fronto-central and centro-parietal electrodes (Figure 3). A marginally non-significant effect of TMS was also found (*P* = 0.05, cluster-corrected).

**Figure 4:**
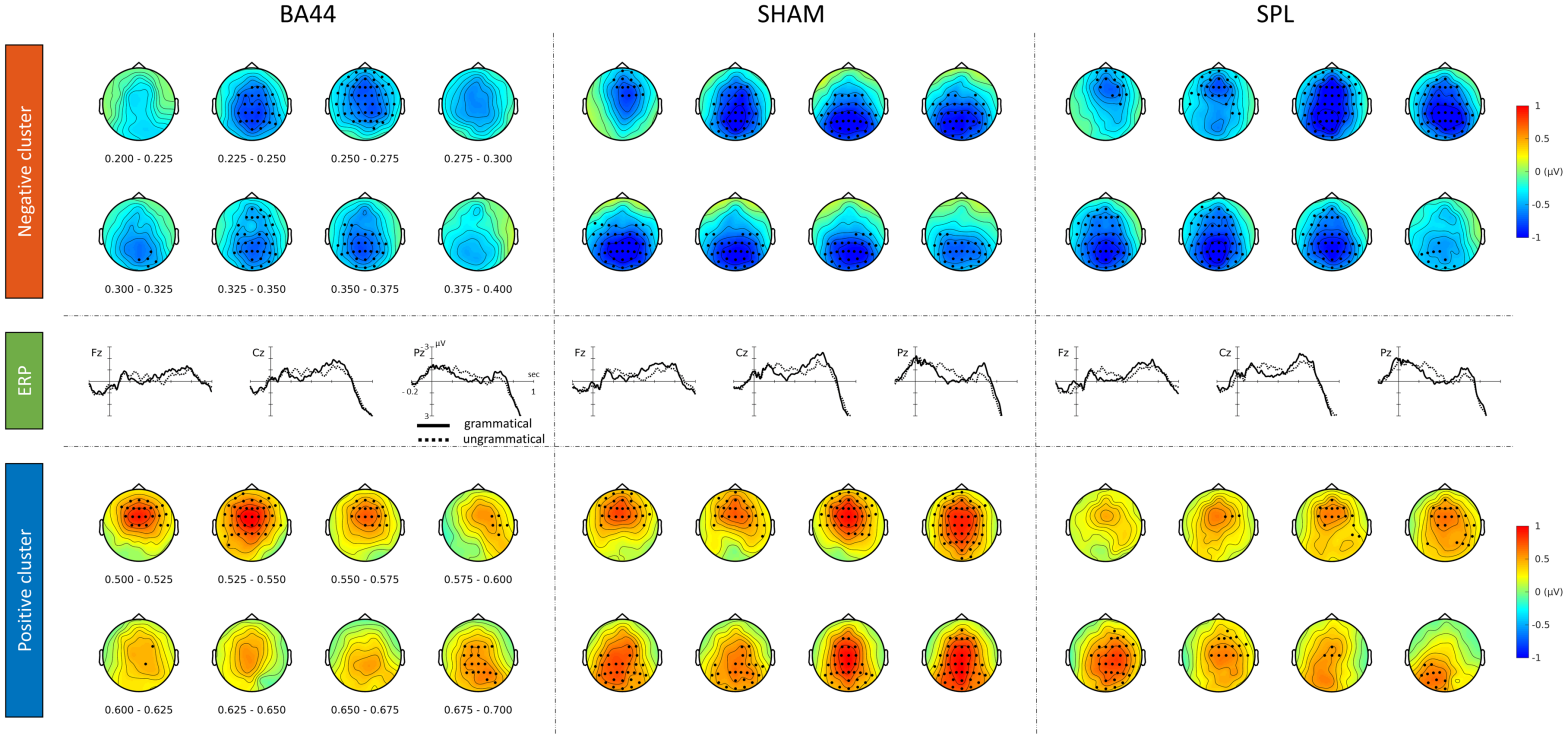
Grammaticality effect within each TMS condition. Electrodes and time-points providing the highest contribution to the significance of the clusters are highlighted.

The Grammaticality × TMS interaction of interest was not significant (*P >* 0.5, cluster-corrected).^9^ The ERP waveforms of grammatical and ungrammatical conditions within each TMS are shown in Figure 4. Additional electrodes are displayed in Supplementary Figures S2-S4. The absence of the interaction is evidenced by the presence of an increased negativity and a late positivity for the ungrammatical condition in each TMS condition. Indeed, within each TMS condition significant clusters were found (BA44: first negative cluster *P* < 0.005, second negative cluster *P* < 0.005, first positive cluster *P* < 0.005, second positive cluster *P* < 0.05; sham: negative cluster *P* < 0.0005, positive cluster *P* < 0.0005; SPL: negative cluster *P* < 0.0005, positive cluster *P* < 0.005). The extent of the clusters in two selected time-windows is shown in Figure 4. The full extent of the clusters within each TMS condition is shown in Supplementary Figures S5-S8. The absence of the critical Grammaticality × TMS interaction shows that TMS over Broca’s area during the first word did not affect the amplitude of the ESN.

Visual inspection of the ERP waveforms of the SPL condition shows an increased positivity for the ungrammatical items approximately 50 ms before the DVP. Crucially, this difference is short-lived, with the waveforms of grammatical and ungrammatical conditions being aligned approximately 30 ms after the DVP. Differences between conditions can be problematic if they are sustained effects and are “masked” by baseline-correction procedure (Steinhauer and Drury, 2012), which we did not perform. A cluster-based permutation test did not reveal a significant difference between conditions in the time-window from -5 to 180 ms relative to the DVP (*P* > 0.5), in line with the non-sustained nature of this difference.

### Bayesian repeated measures ANOVA on the ESN amplitude

The results of the Bayesian repeated measures ANOVA on the ESN amplitude are summarized in Table 3. The best model included only the factors subject and Grammaticality (BF_M_ = 5.651). The model including the Grammaticality × TMS interaction was approximately 10 times less likely than the model with only the main effect of Grammaticality given the data (BF_01_ = 10.295). Direct comparison of the interaction model against the one including the two main effects showed that the former was approximately 6 times less likely given the data (BF_01_ = 6.287).

**Table 3:** Summary of the results of the Bayesian repeated measure ANOVA conducted on the Full ESN.

Models - ESN	P(M)	P(M|data)	BF_M_	BF_10_	BF_01_	Error %
Grammaticality (Gram)	0.200	0.586	5.651	1.000	1.000	-
Gram + TMS	0.200	0.358	2.226	0.611	1.638	2.829
Gram + TMS + Gram × TMS	0.200	0.057	0.241	0.097	10.295	2.985
Null model	0.200	3.145e-9	1.258e-8	5.371e-9	1.862e+8	2.521
TMS	0.200	1.073e-9	4.291e-9	1.832e-9	5.459e+8	2.634

P (M) = prior model probability; P (M|data) = posterior model probability; BF_M_ = posterior model odds; BF_10_ and BF_01_ show the Bayes factors for the comparison of each model against the best one (Grammaticality).

The analysis of the effects is summarized in Table 4. The data are approximately 1.5 × 10^8^ times more likely under models which include the Grammaticality factor (BF_incl_ = 1.539e+8) and two times more likely under models which do not include the TMS factor (BF_excl_ = 2.047). Crucially, the data are four times more likely under models which do not include the Grammaticality × TMS interaction (BF_excl_ = 4.058). Therefore, the additional analysis provide evidence against an effect of TMS over Broca’s area on the amplitude of the ESN component.

**Table 4:** Summary of the analysis of the effects across all models conducted on the Full ESN.

Effects	*P*(incl)	*P*(excl)	*P*(incl|data)	*P*(excl|data)	BF_incl_	BF_excl_
Grammaticality	0.600	0.400	1.000	4.218e − 9	1.581e+8	6.326e-9
TMS	0.600	0.400	0.414	0.586	0.472	2.119
Grammaticality × TMS	0.200	0.800	0.057	0.943	0.241	4.145

P(incl) = prior probability of including a predictor; P(excl) = prior probability of excluding a predictor; P(incl|data) = posterior probability of including a predictor; P(excl|data) = posterior probability of excluding a predictor; BF_incl_ = Bayes factor for including a predictor; BF_excl_ = Bayes factor for excluding a predictor.

### ESN and induced electrical field simulation

The average intensity of the induced electrical fields in each ROI is summarized in Table 5. Within Broca’s area, the average electrical field was higher in BA45 (80.05 V/m) than BA44 (59.68 V/m). Within the SPL, the ROIs in which TMS induced the highest electrical field were BA7PC (52.60 V/m), BA5L (41.66 V/m) and BA7A (41.33 V/m). The reconstructed electrical fields for each subject, mapped to fs average space, are shown in Supplementary Figures S10, S11.

**Table 5:** Mean and standard deviation of the induced electrical field (V/m) in the nine ROIs of interest.

Mean electrical field (SD)			
Broca’s area		Superior parietal lobe	
BA44 BA45	59.68 (12.65) 80.05 (16.10)	BA5Ci	15.76 (3.03)
		BA5L	41.66 (6.69)
		BA5M	17.52 (2.97)
		BA7A	41.33 (7.16)
		BA7M	13.02 (2.44)
		BA7P	23.29 (3.90)	
		BA7PC	52.60 (12.15)

**Figure 5:**
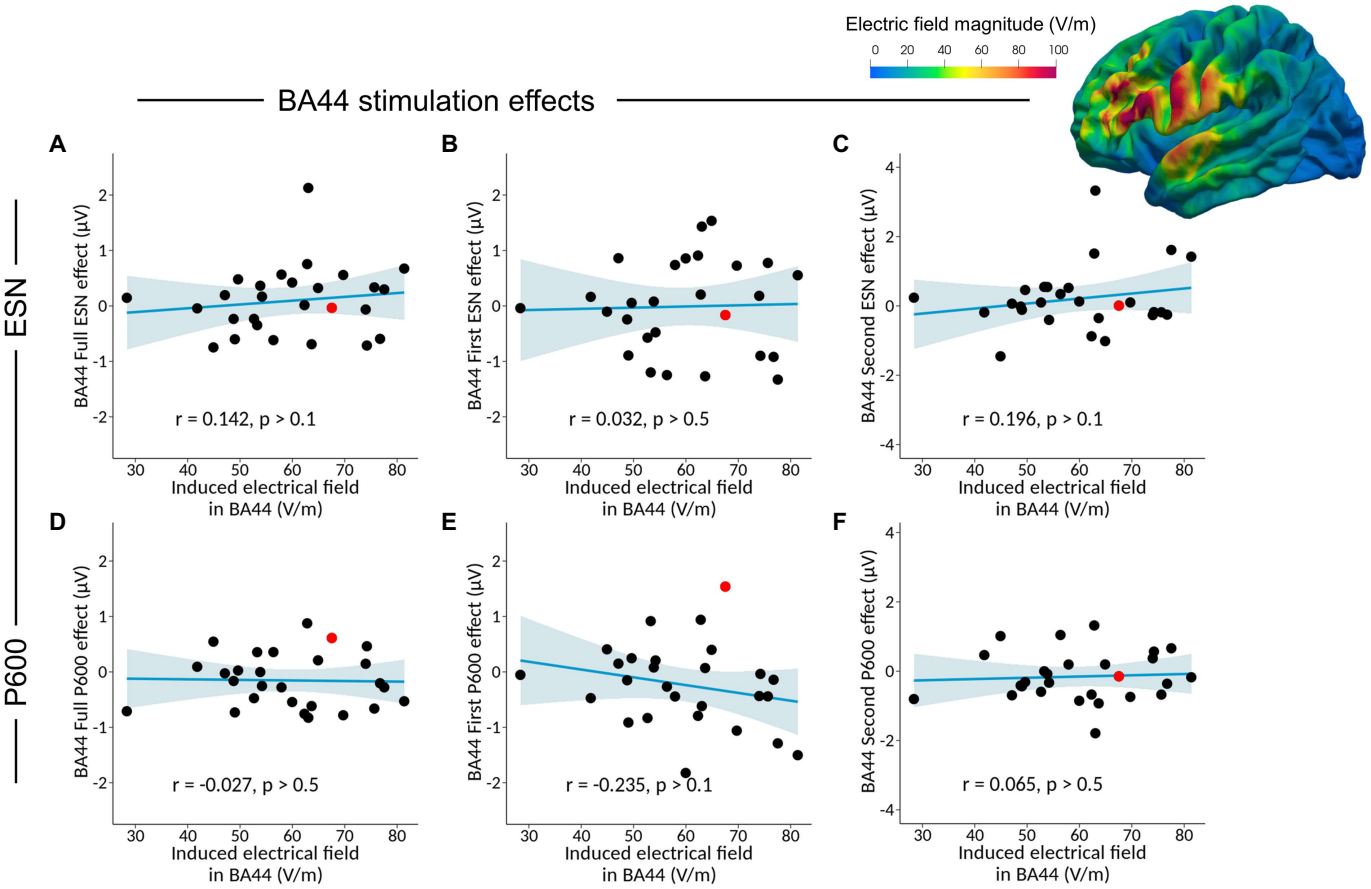
Analysis of the correlation between the Full ESN_BA44_ effect (Full ESN_BA44_ – Full ESN_sham_) and the induced electrical field in BA44 **(A)**, together with separate correlation analyses for the First ESN_BA44_ effect **(B)**, Second ESN_BA44_ effect **(C)**, Full P600_BA44_ effect **(D)**, First P600_BA44_ effect **(E)**, and Second P600_BA44_ effect **(F)**. The plotted brain illustrates the reconstructed TMS-induced electrical field from the BA44 session for a single subject, highlighted in red in the scatter plots.

Considering the Full ESN time-window, no significant correlation was found between Full ESN_BA44_ effect and the induced electrical field in BA44 (Table 6 and Figure 5, *r =* 0.142, *p* > 0.1, BF_01_ = 3.302, with median posterior *δ* = 0.128, 95% Credible Interval CI = [−0.239, 0.473]). The BF_01_ indicates that the data are 3.302 times more likely under the null hypothesis compared to the alternative one. Similarly, no significant correlation was found between Full ESN_BA44_ effect and the induced electrical field in BA45 (*r =* 0.114, *p* > 0.5, BF_01_ = 3.588, with median posterior *δ* = 0.103, 95% CI = [−0.264, 0.452]).

**Table 6:** Analysis of the correlation between the induced electrical field in the subregions of Broca’s area and the three ESN effects of interest.

ESN effect	ROI eField	*r*	*p*	BF_10_	BF_01_
Full ESN_BA44_ effect	BA44	0.142	0.480	0.303	3.302
Full ESN_BA44_ effect	BA45	0.114	0.570	0.279	3.588
First ESN_BA44_ effect	BA44	0.032	0.874	0.242	4.134
First ESN_BA44_ effect	BA45	−0.006	0.975	0.239	4.182
Second ESN_BA44_ effect	BA44	0.196	0.327	0.378	2.648
Second ESN_BA44_ effect	BA45	0.120	0.549	0.283	3.529

Considering the first part of the ESN effect, no significant correlation was found between First ESN_BA44_ effect and the induced electrical field in BA44 (*r* = 0.032, *p* > 0.5, BF_01_ = 4.134, with median posterior *δ* = 0.029, 95% CI = [−0.334, 0.387]) and BA45 (*r* = −0.006, *p* > 0.5, BF_01_ = 4.182, with median posterior *δ* = −0.006, 95% CI = [−0.366, 0.356]).

Finally, considering the second half of the ESN effect, no significant correlation was found between Second ESN_BA44_ effect and the induced electrical field in BA44 (*r* = 0.196, *p* > 0.1, BF_01_ = 2.648, with median posterior *δ* = 0.177, 95% CI = [−0.189, 0.513]) or in BA45 (*r* = 0.120, *p* > 0.5, BF_01_ = 3.529, with median posterior *δ* = 0.109, 95% CI = [−0.258, 0.456]).

No significant correlation was found between Full, First or Second ESN_SPL_ effect and the electrical field induced in any of the SPL ROIs (see Supplementary Tables S4-S6). To summarize the analysis, even when accounting for the induced electrical field and the spatio-temporal profile of our ERP effect, our data show that TMS over Broca’s area did not affect the amplitude of the ESN component when inducing a virtual lesion during the online processing of the first word in our two-word paradigm.

### Late positivity/P600 and induced electrical field simulation

Considering the Full P600 time-window, no significant correlation was found between Full P600_BA44_ effect and the induced electrical field in BA44 (Table 7 and Figure 5, *r =* −0.027 *p* > 0.5, BF_01_ = 4.149, with median posterior *δ* = −0.024, 95% CI = [−0.383, 0.339]). No significant correlation was found between Full P600_BA44_ effect and the induced electrical field in BA45 (*r =* −0.040, *p* > 0.5, BF_01_ = 4.105, with median posterior *δ* = −0.036, 95% CI = [−0.394, 0.327]).

**Table 7:** Analysis of the correlation between the induced electrical field in the subregions of Broca’s area and the three P600 effects of interest.

P600 effect	ROI eField	*r*	*p*	BF_10_	BF_01_
Full P600_BA44_ effect	BA44	−0.027	0.894	0.241	4.149
Full P600_BA44_ effect	BA45	−0.040	0.841	0.244	4.105
First P600_BA44_ effect	BA44	−0.235	0.238	0.463	2.159
First P600_BA44_ effect	BA45	−0.139	0.490	0.300	3.337
Second P600_BA44_ effect	BA44	0.065	0.747	0.251	3.983
Second P600_BA44_ effect	BA45	−0.009	0.964	0.239	4.180

Considering the first part of the late positivity/P600 effect, no significant correlation was found between First P600_BA44_ effect and the induced electrical field in BA44 (*r* = −0.235, *p* > 0.1, BF_01_ = 2.159, with median posterior *δ* = −0.213, 95% CI = [−0.541, 0.153]) and BA45 (*r* = −0.139, *p* > 0.1, BF_01_ = 3.337, with median posterior *δ* = −0.125, 95% CI = [−0.470, 0.242]).

Considering the second part of the late positivity/P600 effect, no significant correlation was found between Second P600_BA44_ effect and the induced electrical field in BA44 (*r* = 0.065, *p* > 0.5, BF_01_ = 3.983, with median posterior *δ* = 0.059, 95% CI = [−0.307, 0.413]) and BA45 (*r* = −0.009, *p* > 0.5, BF_01_ = 4.180, with median posterior *δ* = −0.008, 95% CI = [−0.368, 0.354]). Overall, our data show that TMS over Broca’s area at the predictive stage did not affect the amplitude of the late positivity/P600 component.

No significant correlation was found between Full, First or Second P600_SPL_ effect and the electrical field induced in any of the SPL ROIs (see Supplementary Tables S7-S9).

## Discussion

Lesion studies provide evidence for a causal role of Broca’s area in fast syntactic composition (Friederici et al., 1998, 1999), but leave open the question of whether this region is involved in predicting words’ grammatical categories or integrating them into constituents. In this study, we used online TMS in healthy individuals to test specifically the causal role of Broca’s area in syntactic categorical prediction. State-of-the-art modelling of the induced electrical field (Kuhnke et al., 2020; Weise et al., 2020; Numssen et al., 2021) further quantified the impact of TMS in Broca’s area. The present TMS-EEG data provided two main results. First, a main effect of Grammaticality revealed early automatic (ESN) and late controlled (late positivity) syntactic effects at the two-word level. Secondly, the absence of the critical Grammaticality × TMS interaction indicated that the transient disruption of Broca’s area at the stage of categorical prediction did not affect the generation of the ESN (prediction error, according to a predictive coding perspective), nor late repairing processes (late positivity/P600).

### Early and late main effects of grammaticality

The analysis of the main effect of Grammaticality revealed the presence of the ESN (approximately between 190 and 430 ms), followed by a late positivity (approximately between 440 and 800 ms). The presence of the ESN replicates previous work (Hasting and Kotz, 2008) and provides further evidence for an early analysis of categorical information at the most fundamental two-word level.

The onset latency of the ESN in our experiment (~200 ms) was slightly delayed compared to the original ESN study (Hasting and Kotz, 2008). Crucially, in the original ESN study the grammatical category was marked by the presence or absence of a single phoneme after the DVP (e.g., “Kegel_[DVP]_Ø”, *cone*, “kegel_[DVP]_t”, *bowls*) for the majority of the items. In our experiment, the grammatical category was always marked by a full syllable (e.g., “Fal_[DVP]_ter”, *butterfly*, and “fal_[DVP]_tet”, *folds*), which unfolds over a longer time interval compared to a single phoneme. As a consequence, the detection of the grammatical violation is shifted in time. Another difference is the offset time, as our ESN effect lasted approximately 140 ms longer than the one observed by Hasting and Kotz (2008). There are two possible explanations for this discrepancy. On the one hand, considering that a full syllable and not a single phoneme marks the category in our stimulus set, the delayed offset time could simply be a consequence of the shift in the onset latency of the ESN. On the other hand, the extended duration of our main effect might reflect the concatenation of two processes, indexed by a first anterior negativity (ESN) and a second N400. This pattern has previously been reported for agreement (Barber and Carreiras, 2005; Jakuszeit et al., 2013; Hanna et al., 2014) and marked categorical (Hasting et al., 2007) violations at the two-word level. Our stimuli match agreement violations paradigms with respect to the presence of a suffix indicating whether a given construction is grammatical or not. Therefore, the second negativity (N400) in our dataset could reflect an additional process in which a given suffix is compared against an expected one, which can be used to detect ungrammaticality for categorical violations overtly marked.

The ESN was followed by a late positivity, approximately between 440 and 800 ms. This late positivity aligns well with the profile of the P600 (Osterhout and Holcomb, 1993), indexing repairing and re-analysis processes (Friederici, 2011). At the two-word level, the presence of a late positivity has been reported for agreement (Barber and Carreiras, 2005; Hasting and Kotz, 2008) and categorical (Jakuszeit et al., 2013) violations. The present data converge with these earlier studies, demonstrating that the late syntactic processes observed with longer sentential stimuli can be observed already at the minimal two-word level (see also Maran et al., 2022 for a recent review). Overall, our findings suggest that the recursivity that characterizes syntactic composition (Everaert et al., 2015; Friederici et al., 2017) can be observed at the neurophysiological level, with functionally equivalent processes at the basis of building both minimal phrases and more complex structures.

### No effect of unifocal TMS at the predictive stage on the ESN

We perturbed activity in Broca’s area at the stage of syntactic prediction, by delivering TMS at the onset of a function word. We initially expected that disruption of Broca’s area would have interfered with the formation of a categorical prediction leading to the absence of the ESN effect elicited by an ungrammatical continuation of the utterance. However, our data showed that a unifocal disruption of Broca’s area selectively during the hypothesized categorical prediction phase did not affect early (ESN) or late (late positivity/P600) categorical processes.

In the next section, building on our results and the previous literature, we first propose that Broca’s area builds hierarchical structures by means of bottom-up parsing operations, integrating grammatical categories into constituents rather than predicting them. This alternative account is in line with computational linguistics models (Hale, 2014), recent neuroimaging data (Nelson et al., 2017; Bhattasali et al., 2019) and with theoretical views questioning the role of prediction in language (Huettig, 2015; Huettig and Mani, 2016). We conclude by discussing the possible involvement of compensatory mechanisms within the syntactic predictive network (Hartwigsen et al., 2016; Hartwigsen, 2018).

#### Bottom-up parsing operations in Broca’s area

While our results do not support a causal role of Broca’s area in categorical predictions, they appear to be compatible with the alternative hypothesis that this region might be involved in the fast bottom-up integration of words into syntactic structures. Accordingly, in our experiment the ESN was not affected by TMS over Broca’s area simply because the stimulation occurred before this region was involved in the compositional process, as no syntactic rule could be evaluated on an isolated function word.

A first line of evidence supporting bottom-up syntactic composition in Broca’s area comes from studies which compared sentences and phrases against control conditions containing function words. Maguire and Frith (2004) showed increased activation in Broca’s area pars opercularis for sentences than lists, even if both conditions contained predictive function words. Similarly, Zaccarella and Friederici (2015b) reported increased activity in BA44 not only for two-word pseudo-phrases relative to lists (e.g., “Diese Flirk”, *this flirk*, against “Apfel Flirk”, *apple flirk*), but also for determiner phrases compared to a single determiner (“Diese Flirk”, *this flirk,* against “Diese”, *this*). Converging evidence comes from an fMRI study which investigated categorical violations at the two-word level (Herrmann et al., 2012). Herrmann et al. (2012) observed increased activity in BA44 for categorical violations (*pronoun + noun, *preposition + verb) compared to grammatical items (pronoun + verb, preposition + noun). Crucially, the grammatical and ungrammatical items differed only in whether the second word violated a syntactic rule or not, as in principle potential syntactic predictions triggered by the first word would be present in both conditions. Therefore, the increased activation of BA44 in this experiment might reflect the bottom-up detection of an error, indexing that integration into a constituent is disrupted as no grammatical rule to be applied is found (see also Maran et al., 2022 for a similar discussion). Overall, these studies support the notion that Broca’s area, and specifically BA44, is involved in the bottom-up integration of words into structures, as categorical predictions could be generated also in the control conditions. These results converge on recent electrocorticography and fMRI studies, showing that activity of the left IFG correlates with the number of bottom-up parsing operations during naturalistic comprehension (Nelson et al., 2017; Bhattasali et al., 2019).

Following the hypothesis that Broca’s area processes grammatical rules in a bottom-up fashion, incoming words are temporarily stored in memory until syntactic rules can be applied to combine them into syntactic hierarchies, as suggested by the existence of distinct circuits maintaining words in memory and binding them into hierarchical structures (Makuuchi et al., 2009; Iwabuchi et al., 2019). At the two-word level, such a dissociation might be reflected in the distinct functional profile of the frontal operculum/adjacent insula and BA44 (Zaccarella and Friederici, 2015a,b). The frontal operculum and insula increase in activity as a function of the number of words presented (Zaccarella and Friederici, 2015a,b), while increased activation in BA44 is observed only when a grammatical rule can be applied to combine two elements into a constituent (Zaccarella and Friederici, 2015b).

To the best of our knowledge, no study has directly tested the causal role of Broca’s area in bottom-up syntactic composition. Conceptually, this would correspond to the disruption of Broca’s area during the processing of the second word in our paradigm, which might be technically challenging give the large artifacts in the EEG signal caused by TMS pulses (Rogasch et al., 2013, 2014, 2017; Salo et al., 2020). However, evidence from agreement paradigms suggests that the left IFG might be involved in the bottom-up application of syntactic rules. Increased activity has been observed in the left IFG for agreement violations in designs which subtract prediction-related activations (Carreiras et al., 2010; Heim et al., 2010), and lesions in this region result in the absence of the ESN in this domain (Jakuszeit et al., 2013). Crucially, a behavioural TMS study causally links the left IFG to the bottom-up evaluation of grammatical rules, as stimulation of Broca’s area during the second word (i.e., integration stage) of a two-word phrase causally affects morphosyntactic processes (Carreiras et al., 2012).

The reliance of syntactic composition on bottom-up integration rather than prediction finds further support at behavioural level. In a recent study (Pyatigorskaya et al., 2021), syntactic priming was used to test whether the masked presentation of a prime (i.e., a determiner or a pronoun) could influence the recognition of a target’s grammatical category (i.e., noun or verb), regardless of prime awareness. The authors observed an automatic effect of grammar, with longer RTs to ungrammatical structures compared to grammatical ones. By including an additional baseline condition (i.e., a non-word before nouns and verbs), it was possible to test whether this effect reflected facilitation in processing grammatical structures due to the presence of top-down predictions, or inhibition in encountering an ungrammatical item due to the disruption of the bottom-up integration phase. Crucially, no facilitation was observed, suggesting an absence of top-down predictions during automatic syntactic processing. However, reliable inhibition was observed for ungrammatical prime-target relationships, in line with a disruption of the bottom-up integration phrase. These results converge on the post-lexical and inhibitory nature of syntactic effects observed in similar paradigms focusing on agreement features (Seidenberg et al., 1984; Carello et al., 1988; Friederici and Jacobsen, 1999), possibly pointing towards a general computational feature of the syntactic system.

We wish to point out that this hypothesis does not imply that structural predictions are never generated, but rather that they do not necessarily constitute the automatic mechanism which incrementally builds syntactic structures (Matchin et al., 2017, 2019), at least at the basic two-word level. In this respect, it is noteworthy considering whether predictive coding (Rao and Ballard, 1999; Friston, 2003, 2005; Friston and Kiebel, 2009) provides an adequate framework for syntactic composition. Grammar consists of a set of rules which are not probabilistic but deterministic – either something is correct or not – and which are not defined by the individuals. In other words, grammatical rules constitute a model which is not internal and is not constantly updated, contrary to the processes well described under a predictive coding perspective (Zaccarella et al., 2021). Indeed, automatic syntactic processes treat common and uncommon constructions equally, as long as they are grammatical (Friederici et al., 1996; Pulvermüller and Assadollahi, 2007; Herrmann et al., 2009). Individuals can construct internal probabilistic models of how likely it is that specific syntactic structures will be produced (Kroczek and Gunter, 2017), but the pure and automatic application of grammatical rules might just be a binary process: either something is correct or it is not. A similar dissociation seems to exist at the neural level, both spatially and temporally. For example, speaker-specific probabilistic structural expectations have been linked to late time-windows of syntactic analysis rather than the ELAN one (Kroczek and Gunter, 2021), supported by brain regions located outside of the left-lateralized language network (Kroczek and Gunter, 2020).

On a final note, it has been suggested that Broca’s area processes might be better characterized in terms of general cognitive control and resolution of conflict (Novick et al., 2005, 2010), rather than linguistic or syntactic per se. This notion is currently debated, as dissociations between the networks supporting language specific and domain general processes have been recently discussed (Diachek et al., 2019; Fedorenko and Blank, 2020). At the neuroanatomical level, BA44 has been linked to separate cognitive domains (Clos et al., 2013; Zaccarella et al., 2021), which might differently rely on domain general and language specific processes. Accordingly, language specific and domain general processes might in principle be segregated in different portions of Broca’s area. This represents an interesting research question for future studies, possibly employing highly focal TMS methods (Ueno and Sekino, 2021) to map language specific and domain general processes in Broca’s area.

#### Potential compensatory effects within the syntactic network

Our study was specifically designed to selectively test the functional role of a single area (i.e., Broca’s area) during syntactic processing in language. With the present data at hand, we cannot exclude that unifocal TMS over Broca’s area did not affect the ESN amplitude because the region might be part of a larger network, capable of maintaining efficient processing despite focal disruptions of a node. Compensatory mechanisms at the network level have been observed in previous studies, albeit not focusing on syntactic processing (Sack et al., 2005; O’Shea et al., 2007; Hartwigsen et al., 2012, 2013, 2016; Hartwigsen and Siebner, 2015; Jung and Lambon-Ralph, 2016; for a review, see Hartwigsen, 2018).

According to this view, a potential candidate region for network compensation in syntactic processing could be found in the posterior temporal lobe (pTL). At the structural level, the myelinated dorsal tract connecting this region and the left IFG (Friederici et al., 2006; Papoutsi et al., 2011; Wilson et al., 2011; Skeide et al., 2016; Skeide and Friederici, 2016; Zaccarella et al., 2017b) could provide the neural infrastructure for fast compensatory mechanisms. At the functional level, the pTL has been linked to syntactic processing (Law and Pylkkänen, 2021; Matar et al., 2021), and several studies reported its co-activation with the left IFG during syntactic processing (Tyler et al., 2010; Den Ouden D. B. et al., 2012; Bonhage et al., 2015; Matchin et al., 2017; Schell et al., 2017; Zaccarella et al., 2017a,b; Hultén et al., 2019; Chen et al., 2021b). Furthermore, recent studies provide evidence for a role of the pTL in the active generation of structural expectations, albeit in a task-dependent fashion (Matchin et al., 2017, 2019).

While the structural and functional profile of the pTL makes it a possible candidate for compensatory processes within the syntactic predictive network, lesion data suggest that this might be limited to situations in which Broca’s area is temporarily disrupted, as with online TMS, but not permanently damaged. For example, despite the presence of an intact pTL, no ELAN response is observed in patients with lesion of the left IFG (Friederici et al., 1998, 1999). At present no evidence for the existence of compensatory processes within the syntactic network exists, therefore further studies are needed to test this hypothesis, possibly exploiting the TMS condition-and-perturb approach (Hartwigsen, 2015, 2018) to disrupt functioning at the network level. At present, our study leaves open the possibility that a causal involvement of Broca’s area in categorical prediction can be observed once potential compensatory mechanisms are impeded.

## Limitations

We employed an experimental approach specifically designed to investigate the focal involvement of Broca’s area in categorical prediction. To avoid placebo effects described in the context of TMS studies (Duecker and Sack, 2015), we included both a passive control condition (sham) and an active one (SPL). While necessary to ensure specificity of the TMS effects, this allowed us to include only a single experimental site (Broca’s area) in order to avoid the exposure of our participants to a large number of TMS sessions. Importantly, the available evidence did not point towards any alternative substrate for syntactic prediction. For example, most of the activations observed outside of Broca’s area by Bonhage et al. (2015) might have reflected the increased attentional demands triggered by the jabberwocky condition employed (Diachek et al., 2019). Furthermore, other studies speak against the localization of categorical predictions in these regions. For example, the anterior temporal lobe has been linked to conceptual and semantic processing by a series of MEG (Pylkkänen, 2020) and fMRI studies (Baron et al., 2010; Baron and Osherson, 2011; Graessner et al., 2021b), and the frontal operculum and insulae are not modulated by syntactic hierarchy (Zaccarella and Friederici, 2015a,b). Similarly, the intra-parietal sulcus responds to semantic information in a task-dependent manner, rather than supporting automatic abstract categorical processes (Graessner et al., 2021b).

The estimation of the TMS-induced electrical fields in our participants is a first-time quantification of the realized IFG stimulation. To the best of our knowledge, this new perturbation quantification (Weise et al., 2020; Numssen et al., 2021) has not been used elsewhere in the TMS literature on the left IFG. Future studies targeting syntactic processes in Broca’s area, possibly exploiting novel methods for estimating TMS effects on neural processing (Kuhnke et al., 2020; Weise et al., 2020; Numssen et al., 2021), might provide useful insights, either by replicating the present results or by providing evidence for alternative hypotheses.

Due to the artifacts caused by TMS pulses on the EEG signal during the presentation of the first word, the neural indexes of prediction generation and interaction with the stimulation effect could not be directly quantified. Under the hypothesis that categorical predictions are generated, our ERP components would reflect the checking of the incoming word against such an expectation. This issue could be in principle overcome employing an offline TMS protocol (i.e., before the actual task, Hartwigsen, 2015), in combination with an ERP analysis focusing on the time-window of prediction generation (i.e., the first word). Studies employing such an approach, in combinations with control conditions ensuring a syntactic nature of any potential TMS effect, might provide further insights into the neural indexes of categorical prediction generation.

As shown by the Bayesian analyses, our data provided evidence for the null hypothesis (i.e., no effect of BA44 stimulation at the prediction stage on the ESN and late positivity), which was of “moderate”^10^ strength in most of the cases. Only for the two correlations between the electric field induced in BA44 and the First P600_BA44_ and Second ESN_BA44_ effects “anecdotal” evidence was provided, with BF_01_ being 2.159 and 2.468, respectively. Importantly, the strength of evidence reported in our study converges on the one which is present in the literature in TMS (Kuhnke et al., 2020) and EEG (Nieuwland et al., 2020) studies employing Bayesian statistics. The BFs factors from the present study provide the first estimation of the evidence in favour of the null hypothesis on a continuous scale (Faulkenberry et al., 2020), on which future studies can be based.

On a final note, we wish to point out that debate exists on the degree to which linguistic predictions might stem from the hierarchical structure of language (e.g., Brennan and Hale, 2019) or the linear order of words (e.g., Frank and Bod, 2011). Our study alone does not allow to distinguish between these two types of linguistic prediction, but evidence in the literature supports the notion of abstract syntactic processing when analysing basic two-word constructions (see Maran et al., 2022 for a review). For example, effects of grammaticality have been observed in studies employing pseudo-words (Lukatela et al., 1982, 1983), and early ERP components (e.g., sMMN) reflect the well-formedness of two-word constructions rather than the frequency of co-occurrence of their words (Pulvermüller and Assadollahi, 2007; Herrmann et al., 2009). In our study, we attempted to interfere with linguistic predictive mechanisms by stimulating a target area (BA44) which has been implicated in hierarchical syntactic operations (Goucha and Friederici, 2015; Zaccarella and Friederici, 2015b; Zaccarella et al., 2017a,b; Chen et al., 2021a). Further studies are however needed to test whether predictive processes based on linear order, possibly supported by brain regions not involved in hierarchical processes, can be disrupted with online TMS.

## Conclusion

In this TMS-EEG study we tested whether Broca’s area is causally involved during the potential categorical prediction phase in two-word phrasal/sentential constructions. The present data showed that unifocal perturbation of Broca’s area at the predictive stage did not affect the ERP correlates of basic syntactic composition. Our findings are compatible with the proposal that Broca’s area is involved in bottom-up parsing (Nelson et al., 2017; Bhattasali et al., 2019), with words being integrated into constituents whilst the linguistic stream unfolds. The existence of compensatory mechanisms within the syntactic predictive network may represent an alternative testing ground (Hartwigsen et al., 2016; Hartwigsen, 2018). Future studies addressing these neurocognitive hypotheses are awaited to provide further insights into the mechanisms of incremental linguistic composition.

## Data availability statement

The datasets presented in this article are not readily available because of the restrictions of the consent obtained from the participants of the study. In particular, data can be made available only to collaborators within the European Union and in compliance with the General Data Protection Regulation (EU-GDPR). All collaborators must fully comply to the EU-GDPR. Requests to access the datasets should be directed to maran@cbs.mpg.de.

## Ethics statement

The studies involving human participants were reviewed and approved by the local ethics committee of the University of Leipzig. The patients/participants provided their written informed consent to participate in this study.

## Author contributions

MM: conceptualization, methodology, formal analysis, investigation, data curation, writing - original draft, writing—review and editing, and visualization. ON: formal analysis (electrical field simulations), writing—review and editing, and visualization (electrical field simulations). GH: resources and writing—review and editing. EZ: conceptualization, writing—review and editing, visualization, and supervision. All authors contributed to the article and approved the submitted version.

## Funding

MM was supported by the International Max Planck Research School on Neuroscience of Communication: Function, Structure, and Plasticity and by direct funding from the Department of Neuropsychology (Max Planck Institute for Human Cognitive and Brain Sciences).

## Conflict of interest

The authors declare that the research was conducted in the absence of any commercial or financial relationships that could be construed as a potential conflict of interest.

## Publisher’s note

All claims expressed in this article are solely those of the authors and do not necessarily represent those of their affiliated organizations, or those of the publisher, the editors and the reviewers. Any product that may be evaluated in this article, or claim that may be made by its manufacturer, is not guaranteed or endorsed by the publisher.

## Footnotes

^1^The symbol * is conventionally used in theoretical linguistics to indicate an ungrammatical construction.

^2^Ø denotes a so-called zero form, i.e., an absence of a suffix.

^3^See also https://www.fieldtriptoolbox.org/tutorial/tms-eeg/ for a related discussion.

^4^In the case of the interaction, the independent variable to be considered is the grammaticality effect within each TMS condition, which has three levels: BA44 grammaticality effect, SPL grammaticality effect, sham grammaticality effect.

^5^https://jasp-stats.org/ (Accessed September 19, 2022).

^6^The coordinates used as target for stimulating BA44 (Zaccarella and Friederici, 2015b) lie indeed in the most anterior and ventral part of the region, very close to BA45.

^7^An additional analysis was performed to test whether the participants strategically refrained from predicting upcoming grammatical categories along the experiment. This additional analysis (see Supplementary Materials) did not support the notion of strategic blockage of categorical predictive processes in our task.

^8^We describe the temporal and spatial extent only in approximate terms, as recommended by methodological papers on this topic (Maris and Oostenveld, 2007; Maris, 2012; Sassenhagen and Draschkow, 2019).

^9^An additional analysis focusing separately on determiner phrases and sentences can be found in the Supplementary Materials. Crucially, in neither of the cases the Grammaticality × TMS interaction of interest was significant.

^10^BFs have been related to different degrees of strength of evidence (Keysers et al., 2020; Wagenmakers et al., 2018b): anecdotal (1–3), moderate (3–10), and strong (>10).

## Appendix: Stimulus list

**Table:**

Nouns	Verbs
Angler	angelt
Bader^1^	badet
Bastler	bastelt
Bettler	bettelt
Bieter	bietet
Bummler	bummelt
Dichter	dichtet
Drängler	drängelt
Falter	faltet
Fiedler	fiedelt
Gammler	gammelt
Grübler	grübelt
Heuchler	heuchelt
Jodler	jodelt
Leugner	leugnet
Lüfter	lüftet
Mogler	mogelt
Nörgler	nörgelt
Radler	radelt
Regler	regelt
Schlachter	schlachtet
Schlichter	schlichtet
Schnüffler	schnüffelt
Schwindler	schwindelt
Spender	spendet
Sprinter	sprintet
Stammler	stammelt
Tester	testet
Toaster	toastet
Trödler	trödelt
Trommler	trommelt
Zünder	zündet

^1^As pointed out by an anonymous reviewer, one of the selected nouns (“Bader”) might be considered an archaic term by some people. Accordingly, an additional analysis of the EEG data excluding this noun and the relative verb was conducted (see Supplementary Materials). This analysis showed results similar to the ones including the full stimulus list.
