# Supplementary material for: Online neurostimulation of Broca’s area does not interfere with syntactic predictions: A combined TMS-EEG approach to basic linguistic combination
Source: Front Psychol. 2022 Oct 28;13:968836. doi: 10.3389/fpsyg.2022.968836 (PMC9815778; doi:10.3389/fpsyg.2022.968836)
Supplement: Supplementary file 1 [file Data_Sheet_1.pdf]

Supplementary Material

Online neurostimulation of Broca's area does not interfere with syntactic predictions: A combined TMS-EEG approach to basic linguistic combination

Matteo Maran[1,2*], Ole Numssen[3], Gesa Hartwigsen[3], Emiliano Zaccarella[1]

[1] Max Planck Institute for Human Cognitive and Brain Sciences, Department of Neuropsychology, Stephanstraße 1a, 04103, Leipzig, Germany

[2] International Max Planck Research School on Neuroscience of Communication: Function, Structure, and Plasticity, Stephanstraße 1a, 04103, Leipzig, Germany

[3] Max Planck Institute for Human Cognitive and Brain Sciences, Lise Meitner Research Group Cognition and Plasticity, Stephanstraße 1a, 04103, Leipzig, Germany

*** Correspondence:**

Matteo Maran

maran@cbs.mpg.de

Table of contents

1 Response cue colours 2
2 Bayesian repeated measures ANOVA on the late positivity/P600 amplitude 2
3 ERP and induced electrical field simulation (Full, First and Second P600 estimation). 4
4 Supplementary Figures 5
5 Supplementary Tables 13
5.1 ESN time-window 13
5.2 P600 time-window analysis 14
6 Additional analyses 16
6.1 Analysis excluding the trials with the noun "Bader" 16
6.2 Ruling out strategic control over predictive processes 17
6.3 Separate analyses for opened sentences (S) and determiner phrases (DP) 18

1 Response cue colours

Relative luminance of the red and green colours used as response cues was calculated implementing the formula defined in the Web Content Accessibility Guidelines (WCAG) 2.0 (https://www.w3.org/TR/WCAG20/Overview.html#sRGB):

$L = 0.2126*R + 0.7152*G + 0.0722*B$

R, G and B are calculated as follows:

1. RsRGB = R8bit/255; If RsRGB is smaller or equal to 0.03928, R = RsRGB/12.92, otherwise R = ((RsRGB+0.055)/1.055) ^ 2.4;
2. GsRGB = G8bit/255; If GsRGB is smaller or equal to 0.03928, G = GsRGB/12.92, otherwise G = ((GsRGB+0.055)/1.055) ^ 2.4;
3. BsRGB = B8bit/255; If BsRGB is smaller or equal to 0.03928, B = BsRGB/12.92, otherwise B = ((BsRGB+0.055)/1.055) ^ 2.4.

2 Bayesian repeated measures ANOVA on the late positivity/P600 amplitude

We analyzed the effect of TMS on the late positivity component, which appeared as a late main effect of Grammaticality in our data (see section 3.2) and was previously observed for two-word syntactic violations (Hasting & Kotz, 2008; Jakuszeit et al., 2013). This component has been linked to repairing and re-analysis processes (Hasting & Kotz, 2008; Jakuszeit et al., 2013), similarly to the P600 observed with longer stimuli (Hahne & Friederici, 1999). Interestingly, lesion studies provided conflicting evidence on the causal role of Broca's area in late repairing processes (Friederici et al., 1999; Jakuszeit et al., 2013; Wassenaar & Hagoort, 2005). If the late positivity/P600 is causally dependent on the functional disruption of Broca's area at the predictive stage, we should observe a Grammaticality*TMS interaction when analyzing its amplitude.

For the Bayesian repeated measures ANOVA on the late positivity, we extracted the mean amplitude of the waveforms averaging signal between 440 ms and 800 ms at same 40 electrodes used for the Full ESN analysis, in light of the broad topography of both ERP components. Of note, the broad topography is in line with the data reported by Jakuszeit and colleagues (2013). Henceforth we refer to this as the P600 effect. The time-points included in the P600 analysis are based on the extent of the significant positive cluster observed when analysis the main effect of Grammaticality.

The results of the Bayesian repeated measures ANOVA on the P600 amplitude are summarized in Table S1. The best model included only the factors subject, Grammaticality and TMS ($BF_M = 12.944$). The model including the Grammaticality*TMS interaction was approximately 7 times less likely than the model with only the two main effects of Grammaticality and TMS given the data ($BF_{01} = 7.142$). The analysis of the effects is summarized in Table S2. The data are approximately 31500 times more likely under models which include the Grammaticality factor ($BF_{incl} = 31529.512$) and 4.5 times more likely under models which include the TMS factor ($BF_{incl} = 4.498$). The data are approximately twice more likely under models which do not include the Grammaticality*TMS interaction ($BF_{excl} = 2.087$), not supporting an effect of Broca's area stimulation on the amplitude of the late positivity component.

Models Late Positivity/P600	P(M)	P(M|data)	BF_M	BF_{10}	BF_{01}	Error %
Gram + TMS	0.200	0.764	12.944	1.000	1.000	-
Gram	0.200	0.129	0.593	0.169	5.918	29.668
Gram + TMS + Gram*TMS	0.200	0.107	0.479	0.140	7.142	3.579
TMS	0.200	1.652e-5	6.608e-5	2.163e-5	46241	3.216
Null model	0.200	4.623e-6	1.849e-5	6.052e-6	165244	3.152

Table S1. Summary of the results of the Bayesian repeated measure ANOVA conducted on the Full P600. P(M) = prior model probability; P(M|data) = posterior model probability; BF_M = posterior model odds; BF_{10} and BF_{01} show the Bayes factors for the comparison of each model against the best one (Grammaticality + TMS).

Effects	P(incl)	P(excl)	P(incl|data)	P(excl|data)	BF_{incl}	BF_{excl}
Grammaticality	0.600	0.400	1.000	2.114e-5	31529	3.172e-5
TMS	0.600	0.400	0.871	0.129	4.498	0.222
Grammaticality*TMS	0.200	0.800	0.107	0.893	0.479	2.087

Table S2. Summary of the analysis of the effects across all models conducted on the Full P600. P(incl) = prior probability of including a predictor; P(excl) = prior probability of excluding a predictor; P(incl|data) = posterior probability of including a predictor; P(excl|data) = posterior probability of excluding a predictor; BF_{incl} = Bayes factor for including a predictor; BF_{excl} = Bayes factor for excluding a predictor.

With respect to the TMS factor, the waveforms of the P600 component were less negative for the BA44 condition, and increasingly more negative for the SPL and sham sessions (Figure S9). A post-hoc comparison was conducted, focusing on the TMS factor. As summarised in Table S3, evidence for the alternative hypothesis is observed when comparing BA44 and both the SPL and sham conditions (BF_{10} = 2.356 and 5.551, respectively). Moderate evidence for the null hypothesis is observed when comparing the SPL and sham conditions (BF_{01} = 3.406).

		Prior odds	Posterior odds	BF_{10}	Error %
BA44	SPL	1	2.356	2.356	4.281e-7
BA44	sham	1	5.551	5.551	3.069e-8
SPL	sham	1	0.294	0.294	1.227e-5

Table S3: Summary of the post-hoc comparisons conducted on the Full P600 for the TMS effect.

3 ERP and induced electrical field simulation (Full, First and Second P600 estimation).

Given that the late positivity shows an anterior- to-posterior change in topography, similar to the ESN, we divided this component in two parts:

1. First P600: average of signal from 440 ms to 620 ms at 17 anterior electrodes AF3, AFz, AF4, F5, F3, F1, Fz, F2, F4, F6, FC5, FC3, FC1, FCz, FC2, FC4 and FC6. The time-points included correspond to the first half of the Full P600 effect;

2. Second P600: average of signal from 620 ms to 800 ms at 17 posterior electrodes CP5, CP3, CP1, CPz, CP2, CP4, CP6, P5, P3, P1, Pz, P2, P4, P6, PO3, POz and PO4. The time-points included correspond to the second half of the Full P600 effect.

Full $P600_{SPL}$ effect, First $P600_{SPL}$ effect, Second $P600_{SPL}$ effect were obtained with the same procedure described in the main text for the ESN. The analysis correlating these effects to the induced electrical fields can be found in the main text of the manuscript.

4 Supplementary Figures

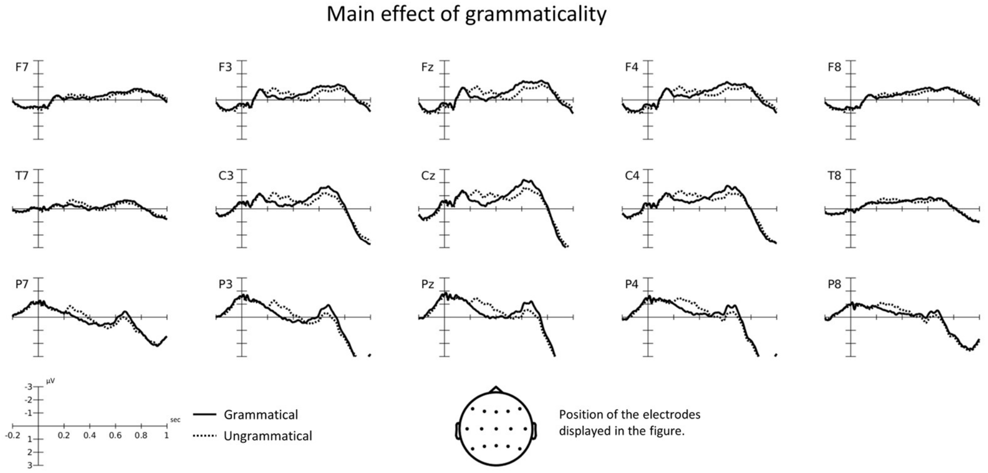

Figure S1: Figure S1 displays the ERP waveforms for the main effect of grammaticality at selected electrodes.

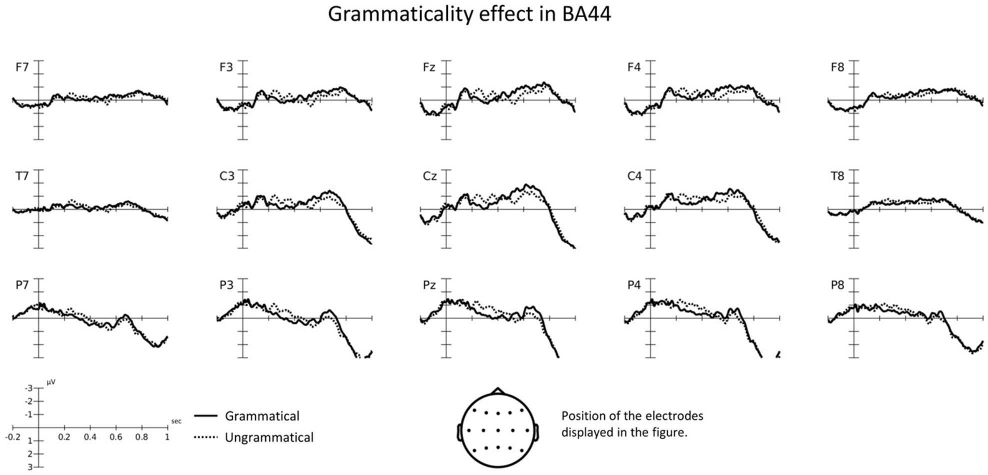

Figure S2: Figure S2 displays the ERP waveforms for the grammaticality effect for the TMS condition BA44 at selected electrodes.

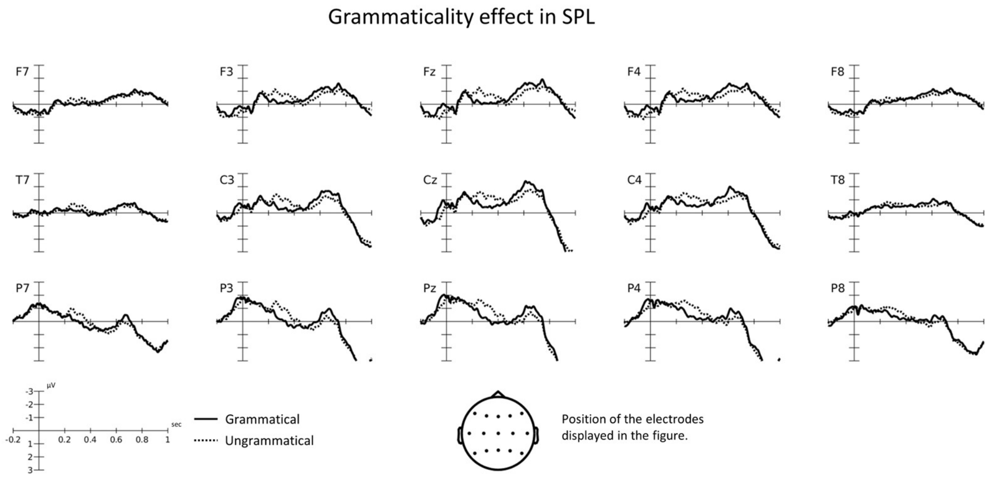

Figure S3: Figure S3 displays the ERP waveforms for the grammaticality effect for the TMS condition SPL at selected electrodes.

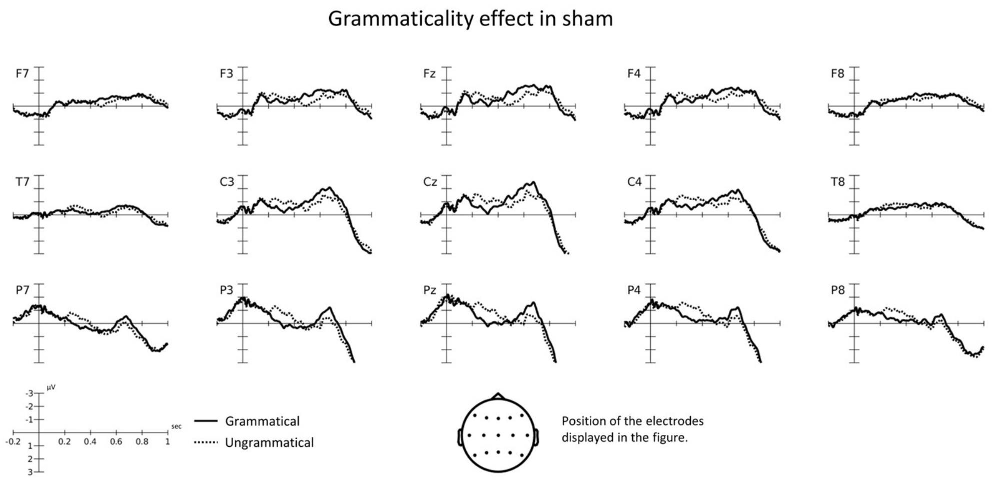

Figure S4: Figure S4 displays the ERP waveforms for the grammaticality effect for the TMS condition sham at selected electrodes.

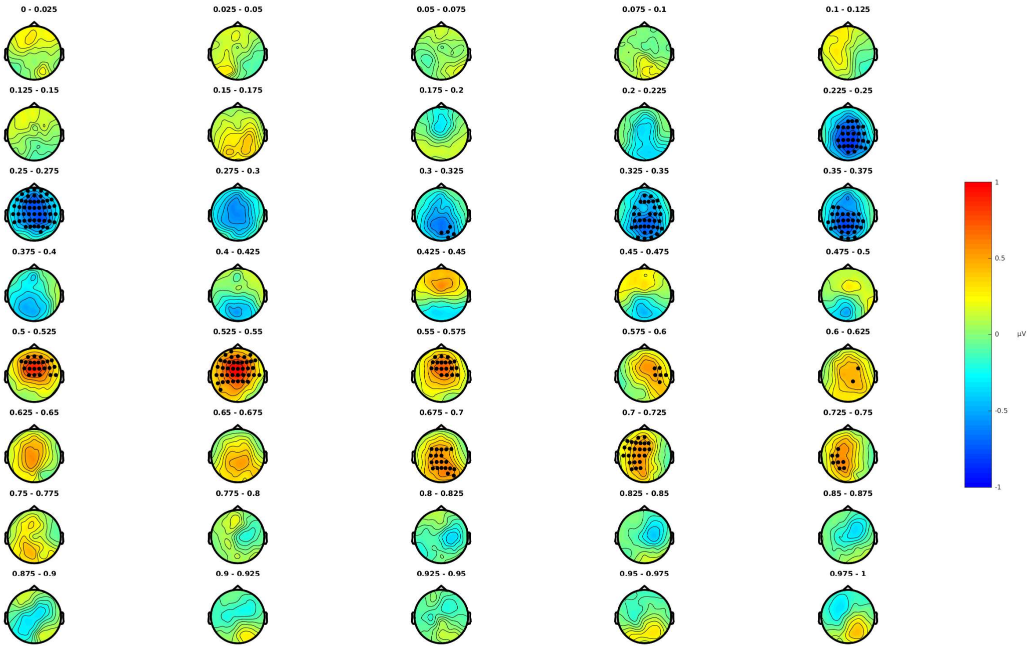

Figure S5: Figure S5 displays the topography of the Grammaticality effect for the BA44 condition. The electrodes and time-points mostly contributing to the significance of the negative and positive clusters are highlighted.

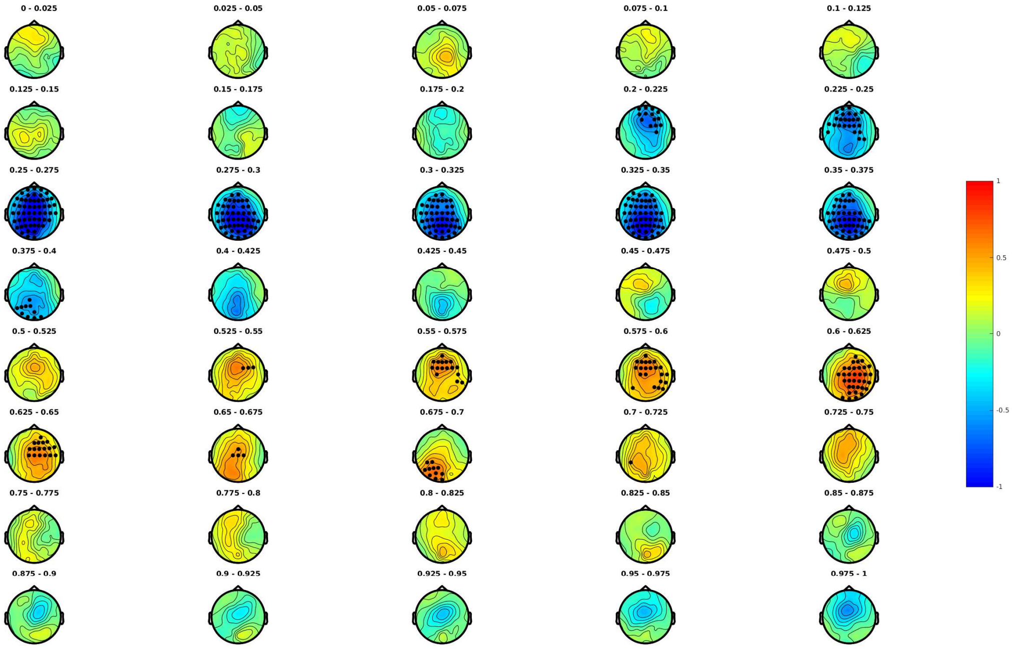

Figure S6: Figure S6 displays the topography of the grammaticality effect for the SPL condition. The electrodes and time-points mostly contributing to the significance of the negative and positive clusters are highlighted.

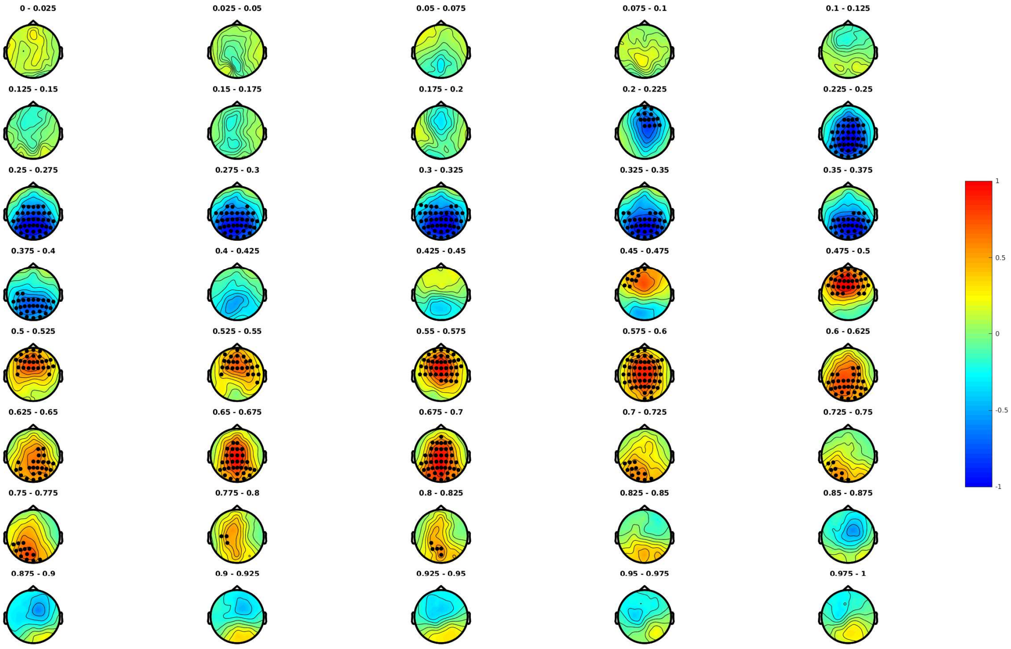

Figure S7: Figure S7 displays the topography of the grammaticality effect for the sham condition. The electrodes and time-points mostly contributing to the significance of the negative and positive clusters are highlighted.

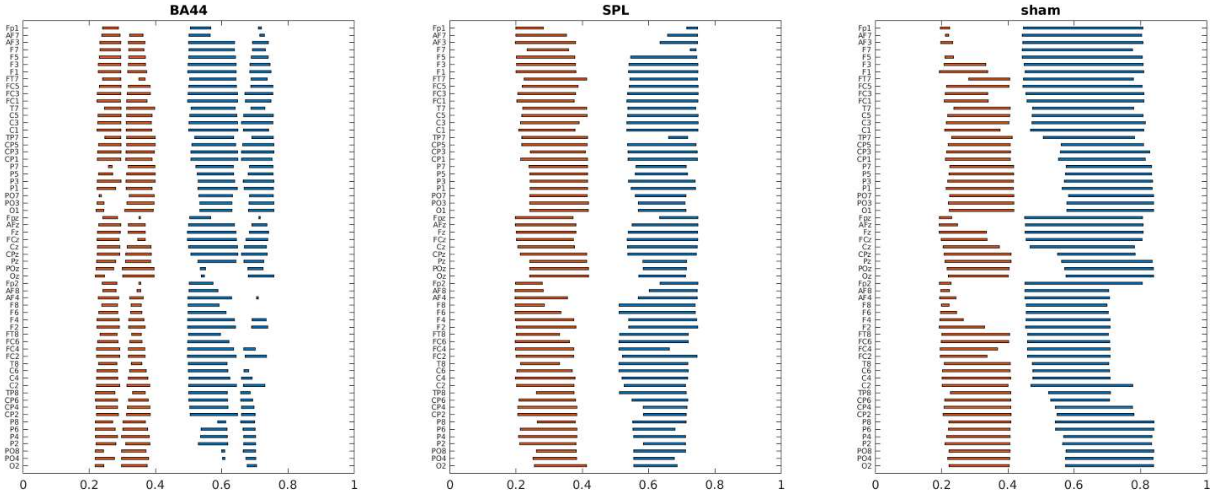

Figure S8: Figure S8 displays the spatial (electrode) and temporal (seconds) extent of the negative (red) and positive (blue) clusters for the Grammaticality effect in each of the TMS conditions.

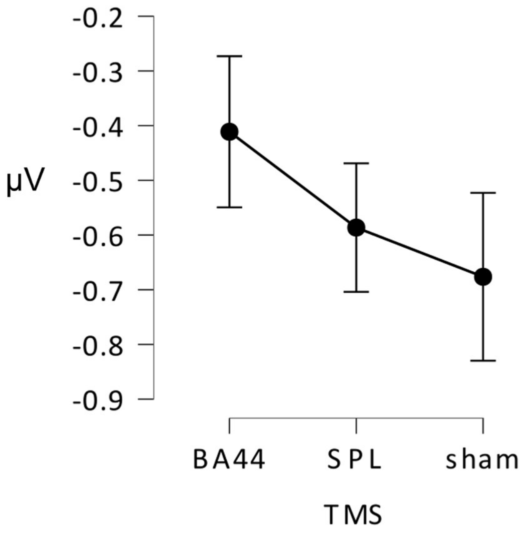

Figure S9: Figure S9 displays the main effect of TMS on the Full P600 ($BF_{incl} = 4.498$), with the bars indicating the 95% credible interval. Figure S9 was generated adapting the output of JASP software (JASP Team, 2020).

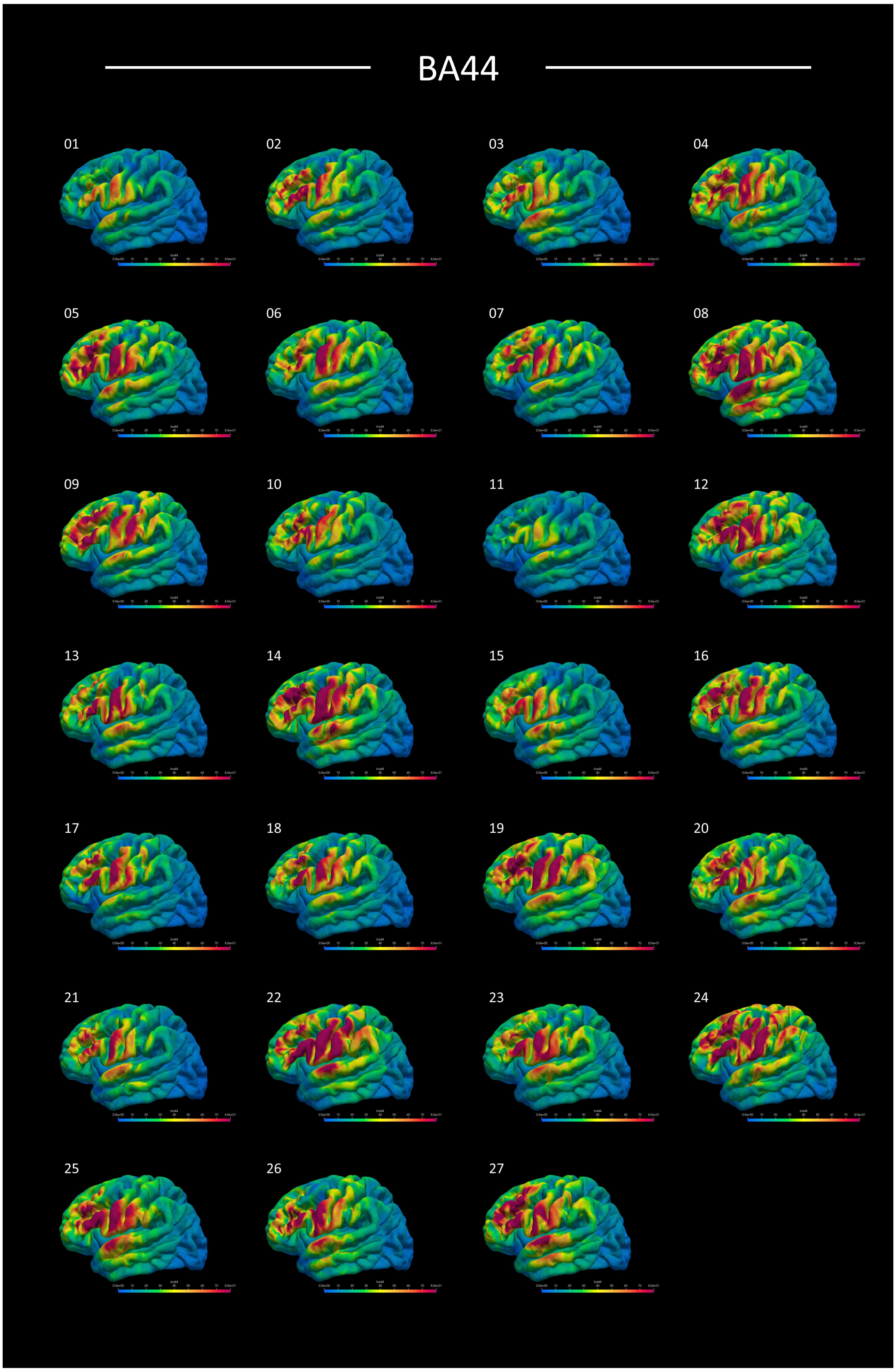

Figure S10: Visualization of the induced electrical field magnitude for the BA44 TMS condition for each of the 27 subjects included in the correlation analysis.

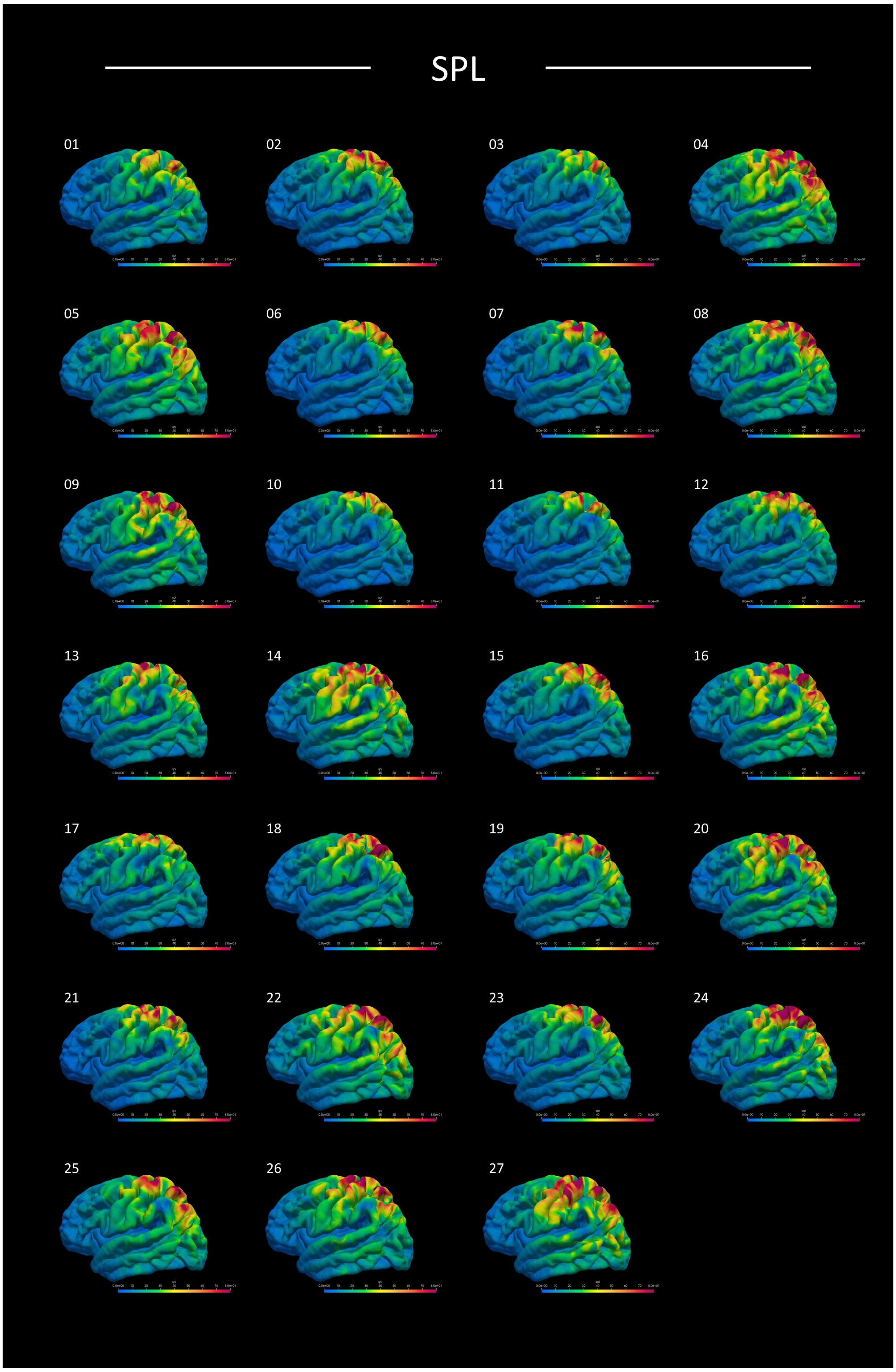

Figure S11: Visualization of the induced electrical field magnitude for the SPL TMS condition for each of the 27 subjects included in the correlation analysis.

5 Supplementary Tables

5.1 ESN time-window

ESN effect	ROI eField	*r*	*p*	BF_{10}	BF_{01}
Full ESN_{SPL} effect	BA5Ci	0.095	0.639	0.265	3.769
Full ESN_{SPL} effect	BA5L	0.261	0.189	0.542	1.845
Full ESN_{SPL} effect	BA5M	0.150	0.454	0.312	3.206
Full ESN_{SPL} effect	BA7A	0.157	0.434	0.320	3.126
Full ESN_{SPL} effect	BA7M	-0.021	0.916	0.240	4.163
Full ESN_{SPL} effect	BA7P	0.089	0.658	0.262	3.812
Full ESN_{SPL} effect	BA7PC	0.011	0.956	0.239	4.178

Table S4: Correlational analysis between the Full ESN_{SPL} effect and the induced electrical field in the subregions of the SPL.

ESN effect	ROI eField	*r*	*p*	BF_{10}	BF_{01}
First ESN_{SPL} effect	BA5Ci	0.104	0.604	0.272	3.682
First ESN_{SPL} effect	BA5L	0.294	0.137	0.685	1.459
First ESN_{SPL} effect	BA5M	0.121	0.546	0.284	3.520
First ESN_{SPL} effect	BA7A	0.149	0.457	0.311	3.217
First ESN_{SPL} effect	BA7M	-0.115	0.569	0.279	3.585
First ESN_{SPL} effect	BA7P	0.079	0.696	0.257	3.891
First ESN_{SPL} effect	BA7PC	-0.008	0.970	0.239	4.182

Table S5: Correlational analysis between the First ESN_{SPL} effect and the induced electrical field in the subregions of the SPL.

ESN effect	ROI eField	*r*	*p*	BF_{10}	BF_{01}
Second ESN_{SPL} effect	BA5Ci	0.124	0.538	0.286	3.493
Second ESN_{SPL} effect	BA5L	0.221	0.268	0.428	2.337
Second ESN_{SPL} effect	BA5M	0.218	0.275	0.421	2.377
Second ESN_{SPL} effect	BA7A	0.172	0.391	0.339	2.949
Second ESN_{SPL} effect	BA7M	0.119	0.555	0.282	3.546
Second ESN_{SPL} effect	BA7P	0.096	0.634	0.266	3.757
Second ESN_{SPL} effect	BA7PC	0.014	0.943	0.240	4.174

Table S6: Correlational analysis between the Second ESN_{SPL} effect and the induced electrical field in the subregions of the SPL.

5.2 P600 time-window analysis

P600 effect	ROI eField	*r*	*p*	BF_{10}	BF_{01}
Full $P600_{SPL}$ effect	BA5Ci	0.082	0.686	0.258	3.871
Full $P600_{SPL}$ effect	BA5L	0.110	0.585	0.275	3.632
Full $P600_{SPL}$ effect	BA5M	0.023	0.911	0.240	4.159
Full $P600_{SPL}$ effect	BA7A	0.069	0.733	0.253	3.960
Full $P600_{SPL}$ effect	BA7M	0.154	0.444	0.316	3.168
Full $P600_{SPL}$ effect	BA7P	0.090	0.654	0.263	3.803
Full $P600_{SPL}$ effect	BA7PC	0.052	0.797	0.247	4.055

Table S7: Correlational analysis between the Full $P600_{SPL}$ effect and the induced electrical field in the subregions of the SPL.

P600 effect	ROI eField	r	p	BF_{10}	BF_{01}
First $P600_{SPL}$ effect	BA5Ci	0.125	0.534	0.287	3.481
First $P600_{SPL}$ effect	BA5L	0.264	0.183	0.556	1.800
First $P600_{SPL}$ effect	BA5M	0.109	0.587	0.275	3.637
First $P600_{SPL}$ effect	BA7A	0.055	0.787	0.247	4.041
First $P600_{SPL}$ effect	BA7M	0.046	0.819	0.245	4.082
First $P600_{SPL}$ effect	BA7P	0.049	0.807	0.246	4.067
First $P600_{SPL}$ effect	BA7PC	0.109	0.588	0.275	3.639

Table S8: Correlational analysis between the First $P600_{SPL}$ effect and the induced electrical field in the subregions of the SPL.

P600 effect	ROI eField	*r*	*p*	BF_{10}	BF_{01}
Second $P600_{SPL}$ effect	BA5Ci	0.136	0.498	0.297	3.362
Second $P600_{SPL}$ effect	BA5L	-0.043	0.831	0.244	4.095
Second $P600_{SPL}$ effect	BA5M	-0.090	0.655	0.263	3.806
Second $P600_{SPL}$ effect	BA7A	0.036	0.857	0.243	4.121
Second $P600_{SPL}$ effect	BA7M	0.291	0.141	0.670	1.493
Second $P600_{SPL}$ effect	BA7P	0.129	0.520	0.291	3.438
Second $P600_{SPL}$ effect	BA7PC	0.155	0.440	0.317	3.153

Table S9: Correlational analysis between the Second $P600_{SPL}$ effect and the induced electrical field in the subregions of the SPL.

6 Additional analyses

6.1 Analysis excluding the trials with the noun “Bader”

An anonymous reviewer pointed out that trials including the word “Bader” might have been associated with anomalous brain responses, since it might be considered an archaic word (denoting a barber or a doctor) by some people. To ensure that this word did not affect the reported results, we conducted repeated measure ANOVAs including and excluding trials with the word “Bader” and the respective verb “badet”. The repeated measures ANOVAs were conducted on the Full ESN and Full P600 amplitudes, calculated as described in the previous section and in the main text. As it indicated in Table S10 and Table S11, the results are unaffected by the presence of “Bader” and “badet” as second words. This probably stems from the low influence of a single trial on the amplitude of the ESN and P600, and from the orthogonal nature of the employed design, in which each word occurs both in grammatical and ungrammatical constructions.

Effect (Full ESN)	DF Num	DF Den	F-value		*p*-value	
			With	Without	With	Without
Grammaticality	1	28	38.643	37.98	<. .001	<. .001
TMS	2	56	2.90	3.12	0.06	0.052
Grammaticality*TMS	2	56	0.83	0.77	0.44	0.46

Table S10: Repeated measures ANOVA on the Full ESN amplitude, including (“With”) and excluding (“Without”) the trials with “Bader” and “badet” as second words.

Effect (Full P600)	DF Num	DF Den	F-value		*p*-value	
			With	Without	With	Without
Grammaticality	1	28	22.57	22.02	<. .001	<. .001
TMS	2	56	2.40	2.35	0.10	0.10
Grammaticality*TMS	2	56	1.25	1.26	0.29	0.29

Table S11: Repeated measures ANOVA on the Full P600 amplitude, including (“With”) and excluding (“Without”) the trials with “Bader” and “badet” as second words.

6.2 Ruling out strategic control over predictive processes

An anonymous reviewer pointed out that the participants might have strategically stopped predicting the upcoming categories since in our design such a prediction would be violated by 50% of the trials. To test this hypothesis, we additionally examined RTs focusing on the first and last trials of the first block. If participants strategically stopped predicting upcoming categories after the first trials of the experiment, the Grammaticality effect (i.e., violation of expectation) should be modulated by the Position in the Block (i.e., first and last trials). In other words, a Grammaticality*Position in Block interaction would be expected.

For each participant, we extracted the first 20 and last 20 trials of the first block from the first TMS session. We then run a linear mixed model analysis using the "lmer" function from the R package "lme4"(Bates et al., 2015), including Grammaticality (grammatical, ungrammatical) and Position in Block (first 20 trials of Block 1, last 20 trials of Block 1) as fixed effects (contrast: sum-to-zero contrast). The random effect structure included a random slope by the Second Word of the item for the main effects of Grammaticality and Position, and a random slope by participant (Subject) for the Grammaticality*Position in Block interaction. More complex random effect structures failed to converge. Log-transformed RTs were used as dependent variable. Accordingly, the following formula for the model was used:

*logRTs~ Grammaticality*PositionInBlock + (1+Grammaticality*PositionInBlock|Subject) + (1+Grammaticality+Position|SecondWord)*

P-values were obtained using the R package "lmerTest" (Kuznetsova et al., 2017) and the "anova" function. As shown in the table below, significant main effects of Grammaticality and Position in Block were observed. Crucially, the Grammaticality*Position in Block interaction was not significant. Accordingly, the data do not support a strategic control over predictive processes.

Effect	Sum Sq	Mean Sq	DF Num	DF Den	F-value	*p*-value
Grammaticality	2.60	2.60	1	28.64	46.08	< .001
Position in Block	1.38	1.38	1	34.12	24.46	< .001
Grammaticality*Position in Block	0.06	0.06	1	27.93	1.16	0.29

Table S12: Type III Analysis of Variance Table with Satterthwaite's method. Sum Sq = Sum of squares; Mean Sq = Mean Squares; DF Num = Degrees of Freedom of Numerator; DF Den = Degrees of Freedom of Denominator.

6.3 Separate analyses for opened sentences (S) and determiner phrases (DP)

As in the previous applications of the ESN paradigm (e.g., Hasting & Kotz, 2008; Herrmann et al., 2012; Jakuszeit et al., 2013), each level of the factor Grammaticality comprises both opened sentences (trials starting with "Er") and determiner phrases (constructions starting with "Ein"). In particular, "Ein + Noun" and "Er + Verb" constructions are analyzed together as "grammatical" structures, while "*Ein + Verb" and "*Er + Noun" as "ungrammatical". This feature of the ESN paradigm ensures that neither the first word ("Ein" or "Er") nor the category of the second one (Noun, Verb) represent a confound for the factor Grammaticality.

An anonymous reviewer pointed out that the type of structure (DP/S) might have interacted with the reported findings. We examined this suggestion in a two-step procedure. First, we inspected whether the grammaticality effect differs between DP and S (averaged across TMS conditions). As displayed below, there are descriptive differences between DP and S:

1. Ungrammatical DPs seem to elicit a larger negativity compared to ungrammatical Sentences
2. Ungrammatical Sentences are characterized by a prolonged negativity, which results in a delayed late positivity.

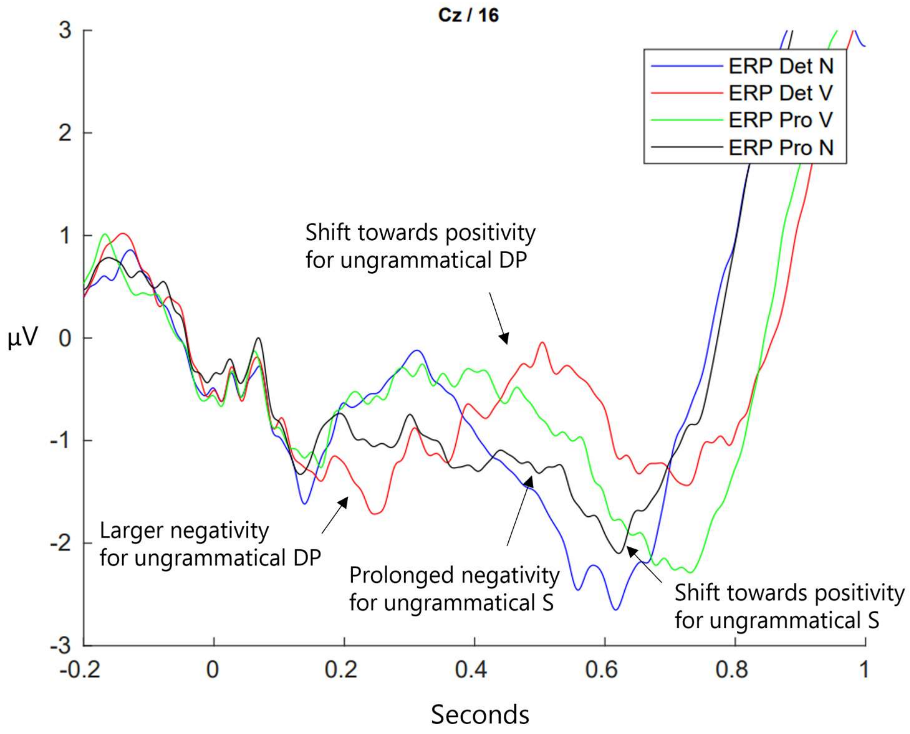

Figure S12: ERP waveforms of the four types of constructions, averaged across TMS. Det = Determiner; N = Noun; Pro = Pronoun; V = verb.

A cluster-based premutation test (1000 permutations) on the Grammaticality*Type of Structure (i.e., DP = Ein + second word; S = Er + second word), run on the time-window ranging from the 0 to 1 second, supported this notion (see Figure S13), revealing the presence of significant clusters: negative ($P < 0.005$; $P < 0.025$) and positive ($P < 0.005$).

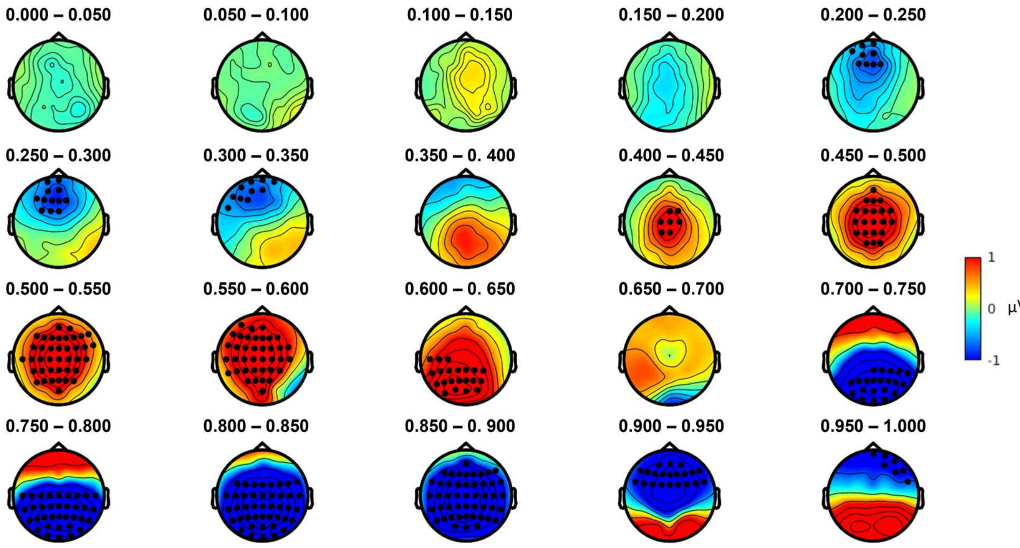

Figure S13: Result of the cluster-based permutation test on the Grammaticality*Structure type interaction. The blue color refers to a larger negativity/decreased positivity for the grammaticality effect in DP (Ein + Verb vs Ein + Noun) compared to S (Er + Noun vs Er + Verb). The red color refers to a larger positivity/decreased negativity for the grammaticality effect in DP compared to S. The electrodes and time-points mostly contributing to the significance of the negative and positive clusters are highlighted.

In a second step, we tested whether a Grammaticality*TMS interaction could be observed when analyzing the two types of structure separately. Accordingly, we run cluster-based permutation tests similar to the ones in the main manuscript ('depsamplesFunivariate', 1000 permutations, latency from 0 to 1 seconds) testing the critical interaction in DP and S structures separately. Crucially, the Grammaticality*TMS interaction of interest was not significant, neither in DP ($P = 0.3$, with $\alpha = 0.025$) nor S ($P = 0.12$, with $\alpha = 0.025$) structures.

References

Bates, D., Mächler, M., Bolker, B. M., & Walker, S. C. (2015). Fitting linear mixed-effects models using lme4. *Journal of Statistical Software*, *67*(1). https://doi.org/10.18637/jss.v067.i01

Friederici, A. D., Von Cramon, D. Y., & Kotz, S. A. (1999). Language related brain potentials in patients with cortical and subcortical left hemisphere lesions. *Brain*, *122*(6), 1033–1047. https://doi.org/10.1093/brain/122.6.1033

Hahne, A., & Friederici, A. D. (1999). Electrophysiological evidence for two steps in syntactic analysis. Early automatic and late controlled processes. *Journal of Cognitive Neuroscience*, *11*(2), 194–205.

Hasting, A. S., & Kotz, S. A. (2008). Speeding up syntax: On the relative timing and automaticity of local phrase structure and morphosyntactic processing as reflected in event-related brain potentials. *Journal of Cognitive Neuroscience*, *20*(7), 1207–1219. https://doi.org/10.1162/jocn.2008.20083

Herrmann, B., Obleser, J., Kalberlah, C., Haynes, J.-D., & Friederici, A. D. (2012). Dissociable neural imprints of perception and grammar in auditory functional imaging. *Human Brain Mapping*, *33*(3), 584–595. https://doi.org/10.1002/hbm.21235

Jakuszeit, M., Kotz, S. A., & Hasting, A. S. (2013). Generating predictions: Lesion evidence on the role of left inferior frontal cortex in rapid syntactic analysis. *Cortex*, *49*(10), 2861–2874. https://doi.org/10.1016/j.cortex.2013.05.014

Kuznetsova, A., Brockhoff, P. B., & Christensen, R. H. B. (2017). lmerTest Package: Tests in Linear Mixed Effects Models. *Journal of Statistical Software*, *82*(13), 1–26. https://doi.org/10.18637/JSS.V082.I13

Wassenaar, M., & Hagoort, P. (2005). Word-category violations in patients with Broca's aphasia: An ERP study. *Brain and Language*, *92*(2), 117–137. https://doi.org/10.1016/j.bandl.2004.05.011
